# Syntenic Relationships between the U and M Genomes of *Aegilops*, Wheat and the Model Species *Brachypodium* and Rice as Revealed by COS Markers

**DOI:** 10.1371/journal.pone.0070844

**Published:** 2013-08-05

**Authors:** István Molnár, Hana Šimková, Michelle Leverington-Waite, Richard Goram, András Cseh, Jan Vrána, András Farkas, Jaroslav Doležel, Márta Molnár-Láng, Simon Griffiths

**Affiliations:** 1 Agricultural Institute, Centre for Agricultural Research, Hungarian Academy of Sciences, Martonvásár, Hungary; 2 Centre of the Region Hana for Biotechnological and Agricultural Research, Institute of Experimental Botany, Olomouc, Czech Republic; 3 Department of Crop Genetics, John Innes Centre, Norwich Research Park, Norwich, United Kingdom; University of Massachusetts Amherst, United States of America

## Abstract

Diploid *Aegilops umbellulata* and *Ae. comosa* and their natural allotetraploid hybrids *Ae. biuncialis* and *Ae. geniculata* are important wild gene sources for wheat. With the aim of assisting in alien gene transfer, this study provides gene-based conserved orthologous set (COS) markers for the U and M genome chromosomes. Out of the 140 markers tested on a series of wheat-*Aegilops* chromosome introgression lines and flow-sorted subgenomic chromosome fractions, 100 were assigned to *Aegilops* chromosomes and six and seven duplications were identified in the U and M genomes, respectively. The marker-specific EST sequences were BLAST-ed to *Brachypodium* and rice genomic sequences to investigate macrosyntenic relationships between the U and M genomes of *Aegilops*, wheat and the model species. Five syntenic regions of *Brachypodium* identified genome rearrangements differentiating the U genome from the M genome and from the D genome of wheat. All of them seem to have evolved at the diploid level and to have been modified differentially in the polyploid species *Ae. biuncialis* and *Ae. geniculata*. A certain level of wheat–*Aegilops* homology was detected for group 1, 2, 3 and 5 chromosomes, while a clearly rearranged structure was showed for the group 4, 6 and 7 *Aegilops* chromosomes relative to wheat. The conserved orthologous set markers assigned to *Aegilops* chromosomes promise to accelerate gene introgression by facilitating the identification of alien chromatin. The syntenic relationships between the *Aegilops* species, wheat and model species will facilitate the targeted development of new markers specific for U and M genomic regions and will contribute to the understanding of molecular processes related to allopolyploidization.

## Introduction

The genus *Aegilops* is closely related to *Triticum* and contains 23 species, 12 of them with U and/or M genomes [1]. The allotetraploid species *Ae. biuncialis* Vis. (2n = 4× = 28, U^b^U^b^M^b^M^b^) and *Ae. geniculata* Roth. (2n = 4× = 28, U^g^U^g^M^g^M^g^), which originated from the natural hybridization of the diploids *Ae. comosa* Sm. in Sibth. & Sm. (2n = 2× = 14, MM) and *Ae. umbellulata* Zhuk. (2n = 2× = 14, UU), have the greatest ecological adaptability [1]. These species represent an outstanding reservoir of useful genes and alleles responsible for tolerance to pests and diseases, including the stem rust strain UG99, and abiotic stresses such as salt, drought, frost and heat stress [2]–[9]. These species have also been reported to carry alleles affecting the nutritional and bread making quality of wheat [10], [11]. These traits are attractive candidates for transfer into bread wheat by interspecific hybridization. Over the past decades, extensive research efforts have been made to introgress *Aegilops* chromatin into wheat, resulting in a range of addition, substitution and translocation lines between bread wheat and *Ae. comosa*, *Ae. umbellulata*, *Ae. geniculata* and *Ae. biuncialis* [2], [3]. As a result of the introgression process, several genes for resistance to rusts and powdery mildew (*Lr9, Lr57, Sr34, Yr8, Yr40, Pm 29*) have been transferred into hexaploid wheat from the U and M genomes of *Aegilops* [2], [3].

The identification of alien chromatin in the wheat genome is an essential part of the pre-breeding process and determines the efficiency of gene transfer efforts. The cytogenetic methods used most commonly to detect *Aegilops* chromatin in the wheat genetic background [2], [12], [13] are powerful techniques, but they tend to be less efficient in identifying small introgressions when the goal is to screen a large population. At the same time, only a small number of cost-effective molecular markers specific for the U and M genomes are available [14]–[17], a fact that limits the high-throughput marker-assisted selection of *T. aestivum - Aegilops* introgression lines. The shortage of suitable DNA markers also slows the development of high density genetic and physical maps, the mapping of favourable agronomic traits and the map-based positional cloning of genes.

Until now, only a few wheat-*Aegilops* translocations have been used in breeding programmes and the introgression of favourable agronomic traits from wild relatives to cultivated wheat remains difficult due to undesirable linkage drag and yield reduction [18], [2]. The utilisation of interspecific translocations in the breeding process is only successful if the introgressed alien chromosome segment compensates for the loss of the wheat chromatin [2]. Compensating wheat-alien translocations are likely to be developed from wheat and alien chromosomes having a strong homoeologous relationship due to the similar gene order along the introgressed chromosome. The collinearity between the homoeologous wheat and *Aegilops* chromosomes could be interrupted by genome rearrangements occurring independently in wheat and *Aegilops* after their evolutionary divergence [19], [20]. For example, Zhang et al. [21] identified at least eleven rearrangements that differentiate the D genome of wheat from that of *Ae. umbellulata.* Therefore, it is extremely important to establish syntenic relationships between the wheat and *Aegilops* chromosomes and to map the breakpoints of genome rearrangements in the U and M genomes relative to wheat.

Rice has been considered as a model system for the *Triticeae* species because of its small genome size (1C = 389 Mb) and the availability of the genome sequence [22], [23]. The comparative mapping of cereal genomes has provided evidence of a high level of conservation of gene order across regions spanning many megabases (i.e. macrocolinearity) [22]. However, the colinearity between rice and *Triticeae* species frequently breaks down at micro level due to translocations, deletions and duplications [24], [25] leading to increased interest in the genome of the wild grass, *Brachypodium distachyon.* This was proposed as a better model organism for structural and functional genomics in cereals because of its biological features (such as self-fertility, inbreeding annual life cycle of less than 4 months, small size, undemanding growth requirements, high capacity for plant regeneration via somatic embryogenesis and resistance to several cereal-adapted pests and diseases), small genome size (1C = 272 Mb) and its closer phylogenetic position to the tribe *Triticeae* [26]–[29]. The genomic sequence of *Brachypodium distachyon* has recently become available [30], allowing a deeper comparison of syntenic regions between crop species and *Brachypodium* as a reference.

Comparative genomic and phylogenetic studies between the *Triticeae*/*Aegilops* taxa and the model systems rice and *Brachypodium* identified a set of genes conserved throughout evolution in both sequence and copy number. This set of >1000 conserved genes, referred to as conserved orthologous set (COS) markers, was identified by the *in silico* comparison of the rice, wheat and Brachypodium EST databases (http://www.wgin.org.uk/resources/Markers/TAmarkers.php; http://www.modelcrop.org/cos_markers) [31]. The COS markers were designed over the exon-intron boundaries of genes conserved between the model and target species. The markers are potentially highly polymorphic, as they span the introns, which have an increased frequency of polymorphisms relative to the exons (6.07 SNP/kb versus 3.00 SNP/kb in introns and exons, respectively, in rice) [32]. These markers define orthologous regions, thus enabling the comparison of regions on the chromosomes of related species. It was shown that COS markers are highly transferable between species such as rice, wheat, maize, sorghum and barley [33]. Wheat-specific COS markers are also transferable to *Aegilops,* as demonstrated by Howard et al. [34], who mapped a major QTL controlling the content of B-type starch granules on chromosome 4S in *Ae. peregrina*. Burt and Nicholson [35] used COS markers to map the eyespot resistance gene *Pch1* originating from *Ae. ventricosa* in hexaploid wheat. Therefore, the COS markers have potential for the identification of alien chromatin introgressed from various species of *Aegilops* into hexaploid wheat, and also to identify the chromosomal locations of orthologous regions in the U and M genomes relative to wheat using rice and *Brachypodium* as references.

The aim of the present study was to assign COS markers to U and M genome chromosomes with the help of a series of wheat-*Aegilops* disomic addition, substitution and translocation lines and using subgenomic DNA samples obtained by flow cytometric sorting of well-defined groups of U and/or M genome chromosomes [17]. A further aim was to compare the *Aegilops* genomes with wheat by identifying orthologous chromosomal regions in the U and M genomes relative to wheat (D genome) using rice and *Brachypodium* as references.

## Materials and Methods

### Plant Materials

The assignment/identification of the COS markers on the U and M genome chromosomes of diploid and allotetraploid *Aegilops* species (*Ae. umbellulata, Ae. comosa, Ae. biuncialis* and *Ae. geniculata*) was carried out on wheat-*Aegilops* introgression lines and on flow-sorted subgenomic DNA fractions with well-defined chromosomal content.

The parental wheat (*Triticum aestivum* L.) genotypes (Chinese Spring, Mv9kr1) of the wheat-*Aegilops* introgression lines and the wheat genotype Mv25, which were used for the first backcross during the production of wheat-*Ae. biuncialis* additions, were used as control. The parental *Aegilops* genotypes of the introgression lines (*Ae. umbellulata* JIC2010001, *Ae. comosa* JIC2110001, *Ae. biuncialis* MvGB642 and *Ae. geniculata* TA2899) and the genoptypes used for the production of flow-sorted subgenomic DNA fractions in previous work [17] (*Ae. umbellulata* MvGB470, *Ae. comosa* MvGB1039, *Ae. biuncialis* MvGB382 and *Ae. geniculata* AE1311/00) were also included in the present study.

The wheat (Chinese Spring)/*Ae. umbellulata* (JIC2010001) addition lines 1U, 2U, 4U, 5U, 6U and 7U, the wheat (Chinese Spring)/*Ae. comosa* (JIC2110001) addition lines 2M, 3M, 4M, 5M, 6M and 7M, and the substitution 6M(6A) were supplied from the John Innes Centre germplasm collection, Norwich, UK by Dr. Steve Reader. The partial set of wheat (Mv9kr1)/*Ae. biuncialis* (MvGB642) addition lines 1U^b^, 1U^b^6U^b^, 3U^b^, 2M^b^, 3M^b^ and 7M^b^ [12], and the substitution 3M^b^(4B) and the centric fusion 3M^b^.4BS, both obtained from a cross between Mv9kr1/*Ae. biuncialis* (MvGB642) 3M^b^ addition × Chinese Spring *ph1b* mutant [36], were produced in Martonvásár. The wheat (Chinese Spring)-*Ae. geniculata* (TA2899) addition lines 1U^g^, 2U^g^, 3U^g^, 4U^g^, 5U^g^, 6U^g^, 7U^g^, 1M^g^, 2M^g^, 3M^g^, 5M^g^, 6M^g^ and 7M^g^ [37] were provided by Dr. Bernd Friebe (Kansas State University, Manhattan, Kansas).

### Chromosome Sorting and Amplification of Subgenomic DNA Samples

Flow cytometric chromosome sorting from individual peaks (I–IV) on flow karyotypes of *Ae. umbellulata* (MvGB470), *Ae. comosa* (MvGB1039), *Ae. biuncialis* (MvGB382) and *Ae. geniculata* (AE1311/00) and the determination of the chromosome content of flow-sorted fractions by FISH were carried out as described by Molnár et al. [17]. The assignment of chromosomes to peaks on flow karyotypes of individual *Aegilops* species is summarized in Table 1. In order to prepare template DNA for PCR with COS markers, chromosomes were sorted in batches of 25–50,000 (equivalent to 20–40 ng) into 40 µl of sterile deionized water in 1.5 ml tubes. The sorted chromosomes were treated with proteinase K and their DNA was amplified by multiple displacement amplification (MDA) using an Illustra GenomiPhi V2 DNA Amplification Kit (GE Healthcare, Chalfont St. Giles, United Kingdom) as described by Šimková et al. [38].

**Table 1:** Chromosome content of subgenomic DNA samples prepared from chromosomes flow-sorted from peaks on flow karyotypes of *Ae. umbellulata* (MvGB470), *Ae. comosa* (MvGB1039), *Ae. biuncialis* (MvGB382) and *Ae. geniculata* (AE1311/00).

Subgenomic DNA samples		Chromosome content*
Species	Flow karyotype peak	
*Ae. umbellulata*	I	1U
	II	6U
	III	3U
	IV	2U, 4U, 5U, 7U
*Ae. comosa*	I	1M, 4M
	II	2M, 6M
	III	2M, 5M
	IV	3M, 7M
*Ae. biuncialis*	I	1U^b^
	II	3U^b^, 6U^b^, 2M^b^, 3M^b^, 4M^b^, 6M^b^
	III	2U^b^, 5U^b^,4U^b^, 7U^b^, 1M^b^, 3M^b^, 5M^b^
	IV	7M^b^
*Ae. geniculata*	I	1U^g^, 6M^g^
	II	3U^g^, 4U^g^, 6U^g^
	III	2U^g^, 5U^g^, 7U^g^, 2M^g^, 4M^g^, 5M^g^
	IV	1M^g^, 3M^g^, 7M^g^

* Chromosomes were assigned to peaks in which they occurred at the highest frequency (Molnár et al. 2011b).

### COS Marker Analysis

DNA preparation and genotyping was carried out as described by Howard et al. [34] using the following templates; wheat-*Aegilops* genetic stocks, parental wheat (Chinese Spring, Mv9kr1, Mv25) and *Aegilops* (*Ae. umbellulata* JIC2010001, *Ae. comosa* JIC2110001; *Ae. biuncialis* MvGB642; *Ae. geniculata* TA2899) genotypes and the *Aegilops* genotypes used for the flow cytometric analysis (*Ae. umbellulata* MvGB470, *Ae. comosa* MvGB1039, *Ae. biuncialis* MvGB382 and *Ae. geniculata* AE1311/00).

A total of 140 markers (whose primer sequences and PCR conditions were summarised in Table S1) potentially covering wheat homoeologous groups I–VII were chosen from two publicly available COS marker collections, the Wheat Genetic Improvement Network (WGIN) (http://www.wgin.org.uk/resources/Markers/TAmarkers.php) and Tools and Resources (TR) collections (http://www.modelcrop.org/cos_markers). When the chromosomal locations of the markers were not available in the D genome of hexaploid wheat, the source EST sequences of the COS markers were searched from the GrainGenes database (http://wheat.pw.usda.gov/cgi-bin/westsql/map_locus.cgi?t=estacc&q=).

Reverse PCR primers were directly labelled with a fluorescent dye (6-FAM) and the following programmes performed on an MJ Research Tetrad PTC-225 Thermal Cycler (Waltham, Massachusetts) were used to amplify PCR products from 10 ng of genomic DNA in 10 µl reactions. WGIN: 95°C (15 min), 39 cycles of (95°C (0.5 min), 58°C (0.5 min), 72°C (0.5 min)), hold at 72°C (5 min) then at 10°C. TR: 94°C (10 min), 16 cycles of (95°C (0.5 min), 58°C (1 min), decreasing by 0.5°C per cycle to 50°C, 72°C (1 min)), 25 cycles of (94°C (0.5 min), 50°C (1 min), 72°C (1 min)), hold at 15°C. The fragment analysis of PCR products was carried out on a POP7 column attached to a 3730×l DNA Analyzer (Applied Biosystems, USA). The results were analysed using GeneMapper v4.0.

### Sequence Analysis

To compare the orthologous regions defined by the COS markers between D genome of *T. aestivum,* U and M genomes of *Aegilops* species and rice and *Brachypodium*, a physical map was constructed showing the physical positions of the COS markers on the chromosomes of rice and *Brachypodium* as reference. To identify the physical positions of the markers, the EST source sequences of the COS markers (shown as Accession No. in Tables S2, S3 and S4) were downloaded from The Institute of Genomic Research (TIGR) database (http://plantta.jcvi.org/index.shtml) and used as queries in BLASTn searches to identify homologues in the assembled genomic sequences of *Brachypodium distachyon* and *Oryza sativa* (*Brachypodium distachyon* v1.0 [30]; Oryza sativa Japonica Group, The IRGSP pseudomolecules, Build 4.0, GenBank Assembly ID: CA_000005425.2) using the EnsemblPlants Database (http://plants.ensembl.org/). As the best hits were considered the hits with the highest score value and characterized by their BLAST parameters E-value, % of Identity and Alignment length (Tables S2, S3 and S4).Throughout the study, BLAST hits with E-values smaller than 2.8e^−08^, Identity % >58.44 and Alignment length >100 bp were considered as significant (Tables S2, 3 and 4).

The start genomic positions of the best hits in *Brachypodium* and rice were used to construct physical maps of the COS markers. The lengths (in bp) of *Brachypodium* and rice chromosomes as well as the start genomic positions of the best hits of the ESTs were converted to pixels and the physical maps of the COS markers were designed.

## Results

### Assignment of COS Markers to U and M Chromosomes

A set of COS markers specific to different ESTs was mapped to *Aegilops* chromosomes using wheat-*Aegilops* introgression lines carrying the U- and M-genome chromosomes of diploid *Aegilops* species (*Ae. umbellulata* and *Ae. comosa*) and their allotetraploid hybrids (*Ae. biuncialis* and *Ae. geniculata*). Subgenomic DNA samples amplified from each of the flow-karyotype peaks of the four goatgrass species and representing individual chromosomes or groups of chromosomes were also used (Table 1).

**Figure 1:**
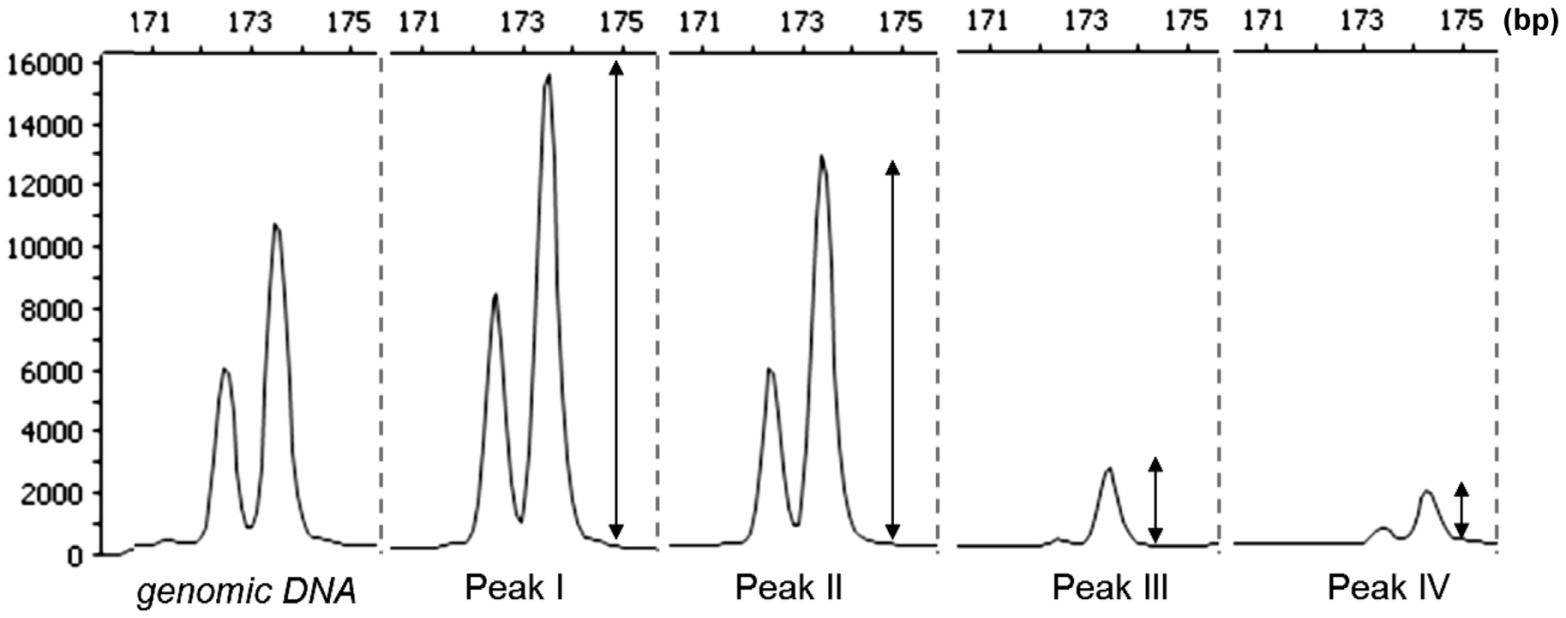
Differences in the yield of a 173 bp PCR product of the *X1N* marker amplified from the genomic DNA and subgenomic DNA samples from peaks I–IV on the flow karyotype of *Aegilops umbellulata* (MvGB470).

Of the 140 COS markers, 133 showed PCR products in the wheat genotypes (Chinese Spring, Mv9kr1 and Mv25) or in at least one of the eight *Aegilops* genotypes, while seven markers did not amplify any product. The 133 markers resulted in 822 PCR products (range: 1–5 PCR products/marker/genotype, mean: 2.04 PCR products; Table S5) with different sizes on the eight genotypes of the four *Aegilops* species, 492 (59.85%) of which showed size polymorphism relative to wheat, while 330 (40.15%) were non-polymorphic. Out of the 492 polymorphic PCR products, 295 (59.95%) products of 89 COS markers could not be unambiguously assigned to *Aegilops* chromosomes because a relevant wheat-*Aegilops* addition line bearing the polymorphic locus was not available and the locus was located in a subgenomic DNA sample representing more chromosomes. 197 (40.04%) polymorphic PCR products were assigned to *Aegilops* chromosomes. The majority of these (159 products) were assigned using wheat-*Aegilops* introgression lines, while the others could be assigned using flow-sorted chromosomes (Table S6). Because each of the *Aegilops* chromosomes has a major location in one of the peaks on a flow-karyotype (Table 1), the yield of PCR products was different on the peak-specific subgenomic DNA samples of the species. Therefore, the highest PCR yield was observed in the peak where the locus-carrying chromosome has its major location (Figure 1). For example, the marker *X1N*, specific for group 1 chromosomes of wheat, produced a 173 bp PCR amplicon with continuously decreasing yield in the *Ae. umbellulata* flow karyotype peaks I, II, III and IV (no amplicon in peak IV) (Figure 1), where the 1U chromosome content was 98.9%, 25.8%, 4.32% and 0%, respectively (Table 1). Based on the yield differences between the subgenomic samples, 25 polymorphic PCR products were also located on *Aegilops* chromosomes, while the genomic positions of 13 fragments were identified simultaneously by introgression lines and by subgenomic DNA samples. Of the 330 non-polymorphic PCR products, 35 could also be assigned using subgenomic DNA samples.

In total, 232 (197 polymorphic and 35 non-polymorphic) PCR products of 100 COS markers were assigned to *Aegilops* chromosomes (Table S6). A significant number of PCR products detected in the tetraploid species *Ae. biuncialis* and *Ae. geniculata* had similar size to the products observed with the corresponding markers in their diploid progenitors. Interestingly, the ratio of non-polymorphic to polymorphic products in the diploid progenitors and their allotetraploid hybrids was higher in the case of the U genome (of the U genome- specific PCR amplicons 80.5% and 77.7% were non-polymorphic in *Ae. biuncialis* and *Ae. geniculata,* respectively, relative to *Ae. umbellulata*) than in the M genome (55.5% and 65.7% in *Ae. biuncialis* and *Ae. geniculata* relative to *Ae. comosa*) (Table S6).

Some markers produced similar sized amplicons both in the diploid progenitors and in the tetraploids *Ae. biuncialis* and *Ae. geniculata*, but the chromosomal location of the locus could not be identified due to the fact that the sets of addition lines were incomplete. When the chromosomal location of a locus could be determined unambiguously in at least one species (in the diploid progenitor, or in *Ae. biuncialis* or *Ae. geniculata*) and the highest product yield in the other two species was detected in the subgenomic DNA sample containing the same chromosome, it was concluded that the locus was located on the same chromosome in all three *Aegilops* species. For example the *X2N* marker produced a 558 bp PCR fragment in *Ae. umbellulata* (AE740/03), *Ae. biuncialis* (MvGB382) and *Ae. geniculata* (TA2899 and AE1311/00), which was also found in the 2U^g^ wheat-*Ae. geniculata* disomic addition line and on the subgenomic samples specific for peaks IV and III of the flow-karyotype containing the 2U and 2U^b^ chromosomes of *Ae. umbellulata* and *Ae. biuncialis*, respectively. As a consequence, it was suggested that the *X2N* marker detects loci on chromosomes 2U and 2U^b^ of *Ae. umbellulata* and *Ae. biuncialis* as well as in *Ae. geniculata.*

Some PCR products could be detected on more than one *Aegilops* chromosome, so 156 loci were assigned unambiguously to the U genome chromosomes. 30 loci (19.23%) were located on group 1 chromosomes, 8 (5.12%) on group 2, 44 (28.20%) on group 3, 21 (13.46%) on group 4, 10 (6.41%) on group 5, 28 (17.94%) on group 6, and 15 (9.61%) on group 7. Out of the 132 loci assigned to the M genome chromosomes, 4 loci (3.03%) were located on group 1 chromosomes, 27 (20.45%) on group 2, 47 (35.60%) on group 3, 3 (2.27%) on group 4, 8 (6.06%) on group 5, 19 (14.39%) on group 6 and 24 loci (18.18%) on group 7 chromosomes. Some markers assigned to *Aegilops* chromosomes showed different chromosomal location in the allopolyploid species relative to the diploid ancestors. The proportion of these markers was significantly higher (27.9%) in the M genome (12 out of 43 markers assigned to chromosomes in *Ae. comosa* and one of the allopolyploid *Aegilops* sp.) than in the U genome (6 of the 74 assigned markers ― 8.1%).

*Aegilops* chromosome-specific markers with a significant level (≥2 bp) of length polymorphism between the parental wheat and *Aegilops* genotypes were considered to be suitable for the marker-assisted selection of new wheat-*Aegilops* introgression lines in prebreeding programmes (Table 2). In this study, 169 polymorphic loci of 51 markers covering all 7 homoeologous groups of the U and M genomes were found to be suitable for the high-throughput detection of diploid and allotetraploid *Aegilops* chromosomes.

**Table 2:** COS markers showing polymorphic (≥2 bp) PCR amplicons between wheat and *Aegilops* species are considered as suitable for the marker-assisted introgression into hexaploid wheat of the U and M genome chromosomes from *Ae. umbellulata* (UU) and *Ae. comosa* (MM) and from *Ae. biuncialis* and *Ae. geniculata*.

	diploid progenitors	*Ae. Biuncialis*	*Ae. geniculata*
1U	*X1B(224), X2B(162)*	*X1B(226), X2B(162), X tr248(208)*	*X1B(226), X2B(162)*
2U	*X2N* * *(558), X2P* * *(292), Xtr146(303), Xtr451(192)*	*X2N* * *(558), Xtr146* * *(303), Xtr451* * *(262)*	*X2N(558), X2P(292), Xtr146(303), Xtr451(262)*
3U	*X3J(205), Xtr62(180), Xtr63(545), Xtr80(429), Xtr83(360),*	*X3J(205), Xtr62(180), Xtr63(545), Xtr77(364),* *Xtr80(429),Xtr83(360)*	*X3J(205), Xtr62(180), Xtr63(545), Xtr80(429), Xtr83(360)*
4U	*X6J* * *(236), Xtr72(179), Xtr76(179), Xtr92(231), Xtr102(318), Xtr103(270)*	*X6J* * *(236), Xtr72* * *(179), Xtr76* * *(179), Xtr92* * *(231), Xtr103* * *(270),*	*X6J(236), Xtr72(179), Xtr76(179), Xtr92(231), Xtr102(318), Xtr103(270), Xtr129(300),*
5U	*X5I* * *(270), X5Q* * *(311), Xtr128(214), Xtr131(470), Xtr248* * *(208)*	*X5I* * *(270), X5Q* * *(311), Xtr128* * *(214), Xtr131* * *(470)*	*X5I(270), X5M(199), X5Q(311), X5S(443), Xtr128(214), Xtr131(470), Xtr248(208)*
6U	*X2I(226), X4C(385), X4G(239), X6A(250), Xtr77(363), Xtr90(290), Xtr91(287), Xtr400(127)*	*X2U(351), X2I(230), X4C(385), X6A(250), Xtr91(287)*	*X4C* * *(385), Xtr90* * *(290), Xtr91* * *(287)*
7U	*X3B* * *(234), X7C* * *(327), X7I(248), Xtr4(266)*	*X3B* * *(234), X7C* * *(327), X7I* * *(248), Xtr4* * *(266)*	*X3B(234), X6A(277), X7C(327), Xtr4(271, 281)*
1M	*X2B* * *(163)*	*X2B* * *(163)*	*X1J(207), X2B(163)*
2M	*X1J* * *(228), Xtr146(381),*	*X1J* * *(228), X2R* * *(267), Xtr72(168), Xtr76(168), Xtr131(356), Xtr134(250),*	*X1J(228), X2I(230), X2R(267),*
3M	*Xtr62(178), Xtr63(444), Xtr67(351), Xtr73(473), Xtr80(487), Xtr83(356), Xtr85* * *(226)*	*Xtr62* * *(178), Xtr63* * *(444), Xtr76(168), Xtr72(168), Xtr80* * *(487), Xtr83* * *(356), Xtr85(226), Xtr131(356), Xtr134(250), Xtr471(263)*	*Xtr62* * *(178), Xtr63* * *(444), Xtr80* * *(487), Xtr83* * *(356), Xtr85* * *(226), Xtr146(381),*
4M	*Xtr88(407)*	*Xtr88* * *(407),*	*Xtr88* * *(407),*
5M	*X5Q* * *(311), Xtr128(212), Xtr471(209), Xtr764(214)*	*X5Q* * *(311), Xtr471* * *(209), Xtr764* * *(214)*	*X5A(245), X5Q(311), Xtr128(210), Xtr471* * *(209), Xtr764(214)*
6M	*X6J* * *(236), Xtr93(477), Xtr103(261), Xtr104(406), Xtr112(390)*	*X6J* * *(236), Xtr103* * *(261),*	*X6J(236), Xtr93(475), Xtr103(261), Xtr104(406)*
7M	*X7C* * *(328), X7I(249, 312)*	*X6A(262), X7C(328), X7I(249, 312)*	*X6A(250), X7C* * *(328), X7I(249, 312)*

The size (in bp) of the chromosome-specific loci is shown in brackets. Asterisks indicate the loci with predicted chromosomal location when the PCR amplicon was specific for the U or M genomes and could be determined unambiguously in at least one *Aegilops* species (in the diploid progenitor, or in *Ae. biuncialis* or *Ae. geniculata*) and when the highest PCR product yield in the other two species was detected in the subgenomic DNA sample containing the same chromosome.

* Loci with predicted chromosomal location.

### Duplications in the U and M Genome of Diploid and Polyploid Aegilops

The chromosomal location of COS markers revealed several intragenomic duplications in the diploid and polyploid *Aegilops* species (Table 3). In the case of the U genome, six duplications were detected. Three (1U/3U, 4U/7U, 6U/7U) were found in the diploid progenitor *Ae. umbellulata* and in one tetraploid *Aegilops*. One duplication (3U/4U) was detected separately in *Ae. umbellulata* and *Ae. geniculata* by markers *Xtr76* and *X7T*, respectively, while two species-specific duplications (1U^g^/2U^g^/7U^g^, 4U^g^/5U^g^) were found in *Ae. geniculata*.

**Table 3:** Duplications in the U and M genomes identified in diploid progenitors (*Ae. umbellulata* and *Ae. comosa*) and in their tetraploid hybrids *Ae. biuncialis* and *Ae. geniculata* by COS markers assigned to *Aegilops* chromosomes.

	Marker	Diploid progenitor	*Ae. biuncialis*	*Ae. geniculata*
U genome	*X6N*	1U/3U	1U^b^/3U^b^	1U^g^/2U^g^/7U^g^
	*Xtr76*	3U/4U	–	3U^g^/4U^g^
	*X7T*	–	–	3U^g^/4U^g^
	*Xtr61*	4U/7U	–	4U^g^/7U^g^
	*X6A*	6U/7U	6U^b^/7U^b^	–
	*X5M*	–	–	4U^g^/5U^g^
M genome	*X1J*	–	–	1M^g^/2M^g^
	*X6N*	–	2M^b^/3M^b^/7M^b^	1M^g^/2M^g^/6M^g^
	*Xtr150*	–	2M^b^/7M^b^	2M^g^/7M^g^
	*X7L*	4M/7M	–	–
	*X7I*	7M/7M	7M^b^/7M^b^	7M^g^/7M^g^
	*X4E*	–	2M^b^/3M^b^	–
	*X4G*	–	2M^b^/3M^b^	–
	*X4I*	–	2M^b^/3M^b^	–
	*X4O*	–	2M^b^/3M^b^	–
	*X4Q*	–	2M^b^/3M^b^	–
	*X4S*	–	2M^b^/3M^b^	–
	*Xtr72*	–	2M^b^/3M^b^	–
	*Xtr76*	–	2M^b^/3M^b^	–
	*Xtr29*	–	2M^b^/3M^b^	–
	*Xtr131*	–	2M^b^/3M^b^	–
	*Xtr134*	–	2M^b^/3M^b^	–

In the M genome, seven different duplications were detected in the diploid (*Ae. comosa*) and the allotetraploids *Ae. biuncialis* and *Ae. geniculata* (Table 3). Some were species- specific, like the 1M^g^/2M^g^ duplication for *Ae. geniculata*, the 4M/7M duplication for *Ae. comosa* and the massive 2M^b^/3M^b^ duplication detected by 11 COS markers for *Ae. biuncialis*. Two duplications were detected in more than one species, such as the 2M/7M duplication in *Ae. biuncialis* and *Ae. geniculata* and the 7M/7M duplication in all the three M genome species.

### Relationship of U and M Genomes Relative to Rice, Brachypodium and Wheat

**Figure 2:**
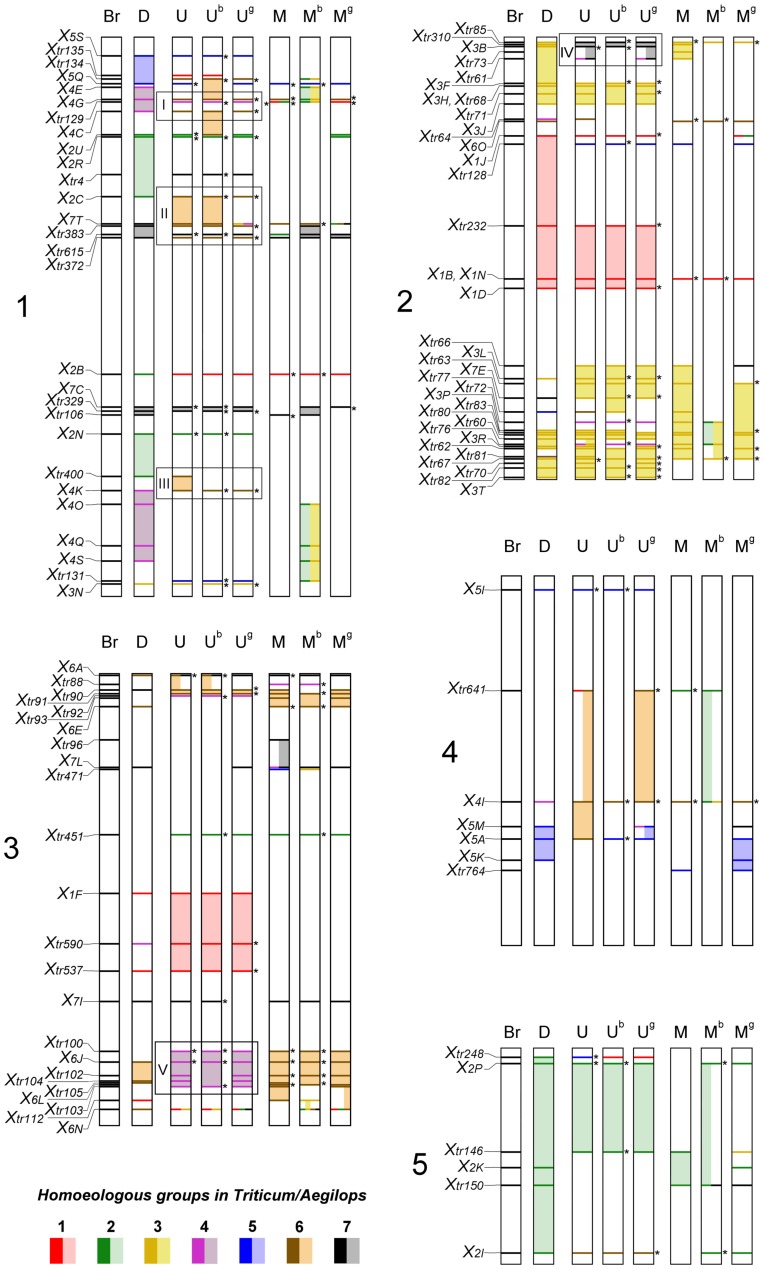
*Brachypodium*–wheat–*Aegilops* orthologous relationships from the genomic perspective of *Brachypodium distachyon*. The physical positions of the source ESTs of the COS markers are indicated on the *Brachypodium* chromosomes (Left). Each marker assigned to chromosomes of the wheat D genome or to the chromosomes of *Ae. umbellulata* (U), *Ae. comosa* (M), *Ae. biuncialis* (U^b^, M^b^) and *Ae. geniculata* (U^g^, M^g^) is colour-coded according to the homoeologous groups of *Triticum*/*Aegilops* chromosomes. Gaps between two markers assigned to the same *Triticum*/*Aegilops* chromosomes are filled in to show synteny (lighter colours). Blocks (designated I–V) indicate *Brachypodium* genomic regions related to the regions in the U genomes involved in evolutionary genome rearrangements relative to the wheat D genome or to M genomes. When a marker mapped to more than one wheat or *Aegilops* chromosome, other colour-coded locations are positioned adjacent to the first one. Asterisks indicate the predicted chromosomal location of a locus when the PCR amplicon was specific for the U or M genomes and could be determined unambiguously in at least one *Aegilops* species (in the diploid progenitor, or in *Ae. biuncialis* or *Ae. geniculata*) and when the highest PCR product yield in the other two species was detected in the subgenomic DNA sample containing the same chromosome.

The source EST sequences of the 100 COS markers identified on the *Aegilops* chromosomes were aligned to the rice and *Brachypodium* sequence databases using BLASTn to identify the genomic positions of the markers in the model species. The genomic distribution of the marker-specific EST sequences in *Brachypodium* and rice and the parameters of the BLAST hits are detailed in Tables S3 and S4. Using the chromosomal length data and the start positions of the best hits, a physical map locating the markers for *Brachypodium* and rice was constructed (Figure 2, Figure S1). Figure 2 provides an overview, from the *Brachypodium* genome perspective, of the genome relationships between the model species and the wheat and *Aegilops* species at the resolution level of the *Trtiticeae*/*Aegilops* chromosomes.

The marker coverage of rice (R) chromosomes R1, R2 and R3 (with 30, 18 and 14 markers per chromosome, respectively) was better than that of the remaining chromosomes (R4–R12). Similar results were obtained for the *Brachypodium* (Br) chromosomes, where 28, 36 and 24 markers were mapped on chromosomes Br1, Br2 and Br3, respectively, while Br4 and Br5 were represented by 7 and 6 markers (Figure 2). The chromosomal locations of the orthologous genes indicated similar structural relationships between the model genomes (*Brachypodium* or rice) and wheat (D genome), as described previously [39], [30]. For example, COS markers specific for wheat (W) chromosome group W3 were located largely on rice chromosome R1, whereas R2 and R3 were generally related to W6 and W4. Moreover, some wheat chromosomes showed homology to two rice chromosomes; for instance, W2 was related to R4 and R7, W1 to R5 and R10 and W7 to R6 and R8. Wheat chromosome 1 was also related to *Brachypodium* chromosomes Br2 and Br3, W2 to Br1 and Br5, W3 to Br2, W4 and W5 to Br1 and Br4, W6 to Br3 and W7 to Br1 and Br3.

In general the homology of the U and M chromosomes of diploid and tetraploid *Aegilops* species to rice and *Brachypodium* was similar to that of wheat (Figure 2, Table 4). Thus, group 3 *Aegilops* chromosomes (Ae) were related mainly to R1 and Br2, whereas the Ae1 chromosomes (1U) showed homology to R5 and R10 and to Br2 and Br3.

**Table 4:** Syntenic genome relationships between the chromosomes of U and M genomes in the diploid progenitors *Ae. umbellulata* and *Ae. comosa* and their allotetraploid hybrids *Ae. biuncialis* and *Ae. geniculata*, and the chromosomes of rice (R) and *Brachypodium* (Br).

	diploid progenitors		*Ae. biuncialis*		*Ae. geniculata*	
	Rice	*Brachy*	Rice	*Brachy*	Rice	*Brachy*
1U	R2*, R3*, **R5**, R7*, R9*, R10, R11*	Br1, **Br2**, **Br3**, Br4*	R2*, R3*, R4*, **R5**, R7*,R9*,R10	Br1, **Br2**, **Br3**, Br5*	R2*, R4*, **R5**, R7*, R9*,R10	Br1*, **Br2**, **Br3**, Br5*
2U	R4, R7, R8*	**Br1**, Br3*, Br5	R4, R7, R8*	Br1, Br3*, Br5	R2*, R4, R7, R8*	**Br1**, Br3, Br5
3U	**R1**, R2*, R6*	Br1*, **Br2**, Br3*	**R1**, R2*, R6*	Br1*, **Br2**, Br3*	**R1**, R6	Br1*, **Br2**
4U	**R1**, **R2**, R3*	Br1*, **Br2**, **Br3**	R1, **R2**, R3*	Br1*, Br2, **Br3**	**R1**, **R2**, R3*, R6*, R9*	Br1, **Br2**, **Br3**, Br4*
5U	**R3**, R4*, R12*	**Br1**, Br2*, Br4*, Br5*	**R3**, R9*, R12*	Br1, Br2*, Br4	**R3**, R9, R12*	**Br1**, Br2*, **Br4**
6U	R1, **R2**, **R3**, R4*, R6, R7, R9*, R11	**Br1**, Br2, **Br3**, **Br4**, Br5*	**R2**, **R3**, R4*, R6, R7, R11*	**Br1**, **Br3**, Br4*, Br5*	R2, **R3**, R4*, R6, R7*, R11	**Br1**, Br3, Br4, Br5*
7U	**R1**, R2*, R6, R7*, R8*, R10*	**Br1**, **Br2**, Br3	R1, R2*, R6, R7*, R8*, R10*	**Br1**, Br2, Br3	**R1**, R2*, R6, R7*, R8*, R10	**Br1**, **Br2**, **Br3**
1M	R3*, R5*, R7*	Br1, Br2*	R5*, R7*	Br1*, Br2*	R2*, R3*, R5, R7*	Br1, Br2**,** Br3*
2M	R3*, R4, R8*, R10*, R11*	Br1, Br3*, Br4*, Br5	**R1**, R2*, **R3**, **R4**, R8*, R11	**Br1**, Br2, Br3, Br4, **Br5**	R2*, **R4**, R5*, R6*, R7*, R8*	**Br1**, Br2*, Br3, **Br5**
3M	**R1**	**Br2**	**R1**, R2, **R3**, R8*, R11*	**Br1**, **Br2**, **Br3**, Br4*	**R1**, R4*	**Br2**, Br4*, Br5*
4M	R2*, R8*	Br3	R2*	Br3*		
5M	R3, R8*, R9*	Br1*, Br2*, Br3*, Br4*	R3*	Br1*	R3, **R9**	Br1*, Br2*, **Br4**
6M	R1*, **R2**, R3*, R6*, R11*	Br1, Br2*, **Br3**, Br4*	R1*, **R2**, R6*	Br1*, Br2*, **Br3**	R1*, **R2**, R3*, R6*, R11*	Br1?, Br2*, **Br3**, Br4*
7M	R2, R6*, R8	Br1, **Br3**	R2, R3*, R4*, R6, R8, R10*	**Br1**, **Br3**, Br5*	R1*, R4*, R6, R8, R9*, R10*	**Br1**, Br2*, **Br3**, Br4*, Br5*

Bold letters indicate rice or *Brachypodium* genomic regions represented by at least three markers.

* :Genomic regions represented by one marker.

Five large-scale chromosomal rearrangements (I–V) identified by more than one marker were detected on the *Aegilops* genomes relative to the wheat D genome and also on the U genome relative to the M genome (Figure 2). The first of these (Rearrangement I), the region spanning from *X4G* to *X4C* on Br1 (or R3) and related to W4, was located on 6U and on various chromosomes of the M genome (6M, 2M^b^–3M^b^, 1M^g^ in *Ae. comosa*, *Ae. biuncialis* and *Ae. geniculata*, respectively). Another Br1 (or R6 and R7) region from *X2C* to *Xtr372* (Rearrangement II), related to W2 (*X2C*) and W7 (*X7T*, *Xtr383*, *Xtr372*), was identified on the group 6 chromosomes of the U genome and also on group 6 (*X7T*) and 7 (*Xtr383-Xtr372*) of the M genome. A further region on Br1 (or R3 and R7), defined by the markers *Xtr400* and *X4K* (Rearrangement III) which are related to W2 and W4, respectively, was also found on the 6U chromosomes of *Ae. umbellulata* and in some cases on the polyploid *Aegilops* species.

The *Xtr85-X3B* region on the distal part of the short arm of Br2 (or R1) (Rearrangement IV), which is related to the group 3 chromosomes of wheat and to the M genomes, was homoeologous with the 7U chromosome in the diploid and polyploid *Aegilops*. The massive region spanning from *Xtr100* to *Xtr103* on the long arm of Br3 (or R2) (Rearrangement V) was homologous with the group 6 chromosomes of wheat and the M genome (in diploid and polyploid *Aegilops* species), whereas it was related to the 4U chromosomes of the three *Aegilops* species. Additional genome rearrangements detected by single markers were also found in the U and M genomes relative to each other and to wheat.

### Syntenic Relationship of U and M Genomes Relative to Wheat

The markers whose EST sequences could be located on wheat allowed the direct investigation of syntenic relationships between wheat and the U and M genomes of *Aegilops*. Table 5 summarises the conserved genomic regions while Table S7 shows the syntenic relationship established based on COS marker positions (Figure 2) between the U and M genomes of diploid and tetraploid *Aegilops* species relative to wheat.

**Table 5:** Conserved genomic regions between the D genome of hexaploid wheat and the chromosomes of the U and M genomes of the diploid species *Ae. umbellulata* and *Ae. comosa* and their allotetraploid hybrids, *Ae. biuncialis* and *Ae. geniculata.*

Group of *Aegilops* chr.	U genome			M genome		
	*Ae. umbellulata*	*Ae. biuncialis*	*Ae. geniculata*	*Ae.Comosa*	*Ae. biuncialis*	*Ae. geniculata*
1	W1 (6)	W1 (6)	W1 (6)	W1 (1)	W1 (1)	W1 (1)
	W2 (1)	W2 (2)	W2 (2)	W2 (1)	W2 (1)	W2 (1)
	W4 (1)	W4 (1)	W4 (1)			
2	W2 (4)	W2 (3)	W2 (4)	W2 (1)	W2 (2)	W2 (5)
3	W3 (15)	W3 (13)	W3 (14)	W3 (6)	W1 (1)	W3 (5)
	W6 (1)	W5 (1)	W7 (1)	W5 (1)	W3 (2)	
	W7 (1)	W7 (1)				
4	W6 (2)	W6 (1)	W6 (2)	–	–	–
5	W2 (1)	W5 (3)	W5 (4)	W5 (1)	W5 (1)	W5 (3)
	W5 (3)					
6	W2 (3)	W2 (3)	W2 (2)	W1 (1)	W6 (4)	W6 (4)
	W4 (5)	W4 (4)	W4 (4)	W4 (2)	W7 (1)	W7 (1)
	W5 (2)	W7 (4)	W7 (3)	W6 (4)		
	W7 (4)			W7 (2)		
7	W3 (2)	W3 (2)	W3 (2)	W6 (1)	W6 (1)	W2 (1)
	W7 (3)	W7 (3)	W6 (1)	W7 (3)	W7 (6)	W5 (1)
			W7 (3)			W6 (1)
						W7 (4)

The number of COS markers representing the wheat orthologous regions is shown in brackets.

## Discussion

### Assignment of COS Markers to U and M Chromosomes

In the present study 94.3% of the COS markers produced amplicons in at least one *Aegilops* species, indicating the high transferability of the conserved orthologous set markers between the related species. The good transferability of COS markers was also reported for *Ae. peregrina* and *Ae. ventricosa* by Howard et al. [34] and Burt and Nicholson [35], respectively. The present results also indicate that the transferability of COS markers is better than other types of molecular markers such as SSRs, where transferability of wheat-specific markers was 80.3% (for *Ae. geniculata*), 79.62% (for *Ae. biuncialis*) and 54.1% for one of the species, *Ae. umbellulata, Ae. comosa, Ae. biuncialis* and *Ae. geniculata* [40], [16], [17]. High transferability between the species could be explained by the sequence conservation of the primer target sites of COS markers, which could be less variable than those of genomic simple sequence repeat markers (SSR).

Wheat SSR markers have been used widely for the molecular characterization of various *Aegilops* species, including *Ae. biuncialis* and *Ae. geniculata* [14], [16], [40]–[42]. Previous studies assigned 33 SSR and 37 sequence-specific amplified polymorphism (S-SAP) markers to U and M chromosomes [15]–[17], [40]. This work significantly increased the number of U and M genome-specific markers by identifying the *Aegilops-*specific chromosomal location for 100 COS markers. One hundred and sixty nine loci of 51 markers covering all 7 chromosomes of the U and M genomes resulted in polymorphic amplicons relative to wheat, so they are potentially useful markers for detecting *Aegilops* chromosomes in bread wheat.

The results also confirmed previous observations on the suitability of MDA-amplified chromosomal DNA for molecular marker analysis [38], [17] and indicate that flow-sorted chromosomes can be used for the physical mapping of molecular markers, especially when a complete set of cytogenetic stocks representing the whole chromosome complements is not available. Furthermore, the possibility of purifying chromosomes in *Aegilops* species [17] opens a way for next-generation survey sequencing to identify low-copy and genic sequences for the development of new markers, including SSR, ISBP, COS and SNP, for genotyping by sequencing of different accessions and for the high-resolution analysis of synteny and the characterization of structural chromosome differences between wheat and its progenitors and relatives [43], [44].

### Relationships between the Genomes of Diploid and Tetraploid Aegilops Species

The theory of pivotal–differential evolutionary patterns in *Aegilops* species suggested by Zohary and Feldman [45] states that the pivotal U genomes remain essentially unchanged during allopolyploid speciation, while the differential M genomes have accumulated substantial modifications as compared with the parental genome [45]–[47]. Consistently with this theory, the inactivation of major NORs on the 1M and 6M chromosomes, the redistribution of 5S rDNA sites, and the loss of minor 18S–26S rDNA loci were observed in *Ae. geniculata* and *Ae. biuncialis* relative to *Ae. comosa* [48], [49], [12], [20]. The amplification, elimination and redistribution of highly repetitive DNA sequences after allopolyploidisation were also more pronounced in the M genome than in the U genome [49], [20]. In the present study, 77.7% and 80.5% of the amplicons assigned to the U genome were non-polymorphic in the allopolyploid species *Ae. geniculata* and *Ae. biuncialis* relative to the diploid progenitor *Ae. umbellulata,* a ratio higher than that observed for M genome loci, which remained unchanged in the allopolyploid species (55.5% in *Ae. biuncialis* and 65.7% in *Ae. geniculata* relative to *Ae. comosa*). These results suggest that, besides the structural chromosomal modifications observed at the macro level, micro level changes may also have happened more frequently in the M genome during or after allopolyploid speciation. The intron regions of the genes, which show a higher frequency of polymorphism in the M genome relative to the U genome, might also be involved in genomic changes related to allopolyploidization.

Little is known about the underlying mechanisms of selective alterations in M-genome chromosomes in (allo)polyploid species. Segmental and single gene duplications were reported to play an important role in the evolution of polyploid species, permitting the functional diversification of paralogues leading to plant adaptation and speciation [50]. In the present study, the number of duplications which were absent in diploid ancestors was higher in the M genomes of *Aegilops* allotetraploids (five duplications detected by fifteen markers) than in the U genomes (three duplications detected by three COS markers) (Table 3). These results suggest that the molecular process of gene duplication is more frequent in the M genome than in the U genome in allotetraploid *Aegilops* species. Two mechanisms were proposed for gene duplication which are linked to the activity of transposable elements (TE) [51], [52]. According to Wicker et al. [52] genes can be captured and copied together with a transposable element to a new location, which has also been documented in allopolyploid plant species [53]. The other mechanism is double-strand break repair involving synthesis-dependent strand annealing, which accompanies the TE insertion [51]. However, earlier studies on the presence of SINE elements in diploid and tetraploid *Aegilops* species with U and M genomes did not support the role of transposable elements in the selective alteration of M genomes during allopolyploidization [15]. The possibilities that allopolyploidization triggered the loss of duplicated genes more frequently in the U genomes, leading to the observation of an apparently lower number of duplicated loci, cannot be excluded. Clearly, a more detailed investigation of allopolyploidization-related changes in the U and M genome chromosomes is needed to explain the distinct difference in the number of duplicated loci on the U and M genomes and to obtain information on the molecular mechanism of their selective alteration.

### Relationships between *Aegilops*, Model Species and Wheat

Previously, wheat-*Ae. umbellulata* macrosynteny was investigated by mapping wheat RFLP markers on the U genome chromosomes of *Ae. umbellulata* [21], [54], [55]. The present work extended the comparative analysis of wheat and *Aegilops* genomes to the M genome of diploid *Ae. comosa,* allowing the U and M genomes in polyploid *Ae. biuncialis* and *Ae. geniculata* to be investigated in relation to wheat, *Brachypodium* and rice. The wheat-*Brachypodium* and wheat-rice genome relationships obtained by the physical location of marker-represented orthologue genes on *Brachypodium* and rice were consistent with previous data reported on syntenic relationships after the sequencing and assembly of the genomes of *Brachypodium* and rice [30], [23]. The physical maps allowed the detection of macrosyntenic relationships between *Aegilops* and the model species. In this respect, previous results indicated that rice chromosome 10 (R10) was inserted into R5 to form *Triticeae* chromosome 1, R7 was inserted into R4 to form *Triticeae* chromosome 2, and R8 was inserted into R6 to form *Triticeae* chromosome 7 [56], [50]. These chromosomal rearrangements were also detected in wheat and in ryegrass chromosomes 1 and 7 [50], [57]. In the present study, the relationship between the R5-R10-R5 insertion and chromosome 1U was also detected, but it was not confirmed for chromosome 1M, where mainly R5 and R7 regions were present (Table 4). In the case of group 2 *Aegilops* chromosomes, the R4-R7-R4 insertion was indicated on 2U chromosomes, but the 2M chromosomes were related mainly to R4 and R8. Finally, the R6-R8-R6 insertion was detected for both the 7U and 7M chromosomes. These results suggest that genome rearrangements derived from common ancestors appear to characterize the U genome of the *Aegilops* species, but were only partly valid for the M genomes.

From an agronomic point of view, the macrosyntenic relationships between *Aegilops* and the model species provide useful background information for the targeted development of markers specific for the *Aegilops* chromosome regions responsible for important agronomic traits [35].

Physical maps of COS markers also allowed the investigation of relationships between the U and M genomes and between wheat and *Aegilops* species in the genomic perspective of *Brachypodium* and rice. Besides the relatively close relationship between the U and M genomes, the chromosomal location of *Brachypodium* and rice syntenic regions in wheat and *Aegilops* genomes detected five genome rearrangements differentiating the U and M genomes. All of them seem to have evolved at the diploid level and to have been modified differentially in the polyploid species *Ae. biuncialis* and *Ae. geniculata*. Three rearrangements (I, II and III) were connected to chromosome 6U, one (IV) to 7U and one (V) to 4U. Interestingly, in three of the five rearrangements (II, IV and V), the genomic regions involved were located on chromosomes in the same homoeologous group in wheat and in the M genomes of *Ae. comosa*, *Ae. biuncialis* and *Ae. geniculata*, while they were located on different homoeologous group chromosomes in the U genome of diploid and polyploid *Aegilops* species. These results suggest that the M genome is more closely related to the wheat D genome than the U genome.

The relationship between the U genome of *Ae. umbellulata* and the D genome of wheat was investigated by Zhang et al. [21], who mapped 79 wheat RFLP markers on wheat cv. Chinese Spring–*Ae. umbellulata* addition lines and 68 on an *Ae. umbellulata* segregating mapping population. This work revealed at least eleven rearrangements that differentiate the D genome of wheat from that of *Ae. umbellulata* [21]. Using a comparable level of marker resolution, the present study generally showed similar U-D homologous relationships to those reported in the previous work. It was found that 1U was related mainly to W1, though the presence of small fragments related to W2 and W4 was also indicated. The relationship of chromosome 1M to W1 and W2 was also detected, and chromosomes 2U and 2M were again found to be related to W2. Zhang et al. [21] showed that 3U was closely related to W3 and suggested the presence of a fragment related to W7 but no experimental evidence was presented. The close relationship of W3 to 3U, and also to 3M, was supported by the present work. Moreover, one marker indicated the presence of a fragment related to W7, which is consistent with the results of Yang et al. [58], who suggested that a translocation may exist between *Ae. umbellulata* chromosomes 3U and 7U. Due to the low number of markers, relationships could only be detected between 4U and W6 and between 5U/5M and W5. The present results confirmed the highly rearranged structure of chromosome 6U and indicated a rearranged structure for the 6M chromosomes for the first time. At the diploid and tetraploid levels, fragments related to W2, W4 and W7 were detected on 6U, as also reported by Zhang et al. [21]. Two markers also indicated the homology of *Ae. umbellulata* 6U with W5, but due to the low number of markers, no experimental evidence could be found for the presence of a fragment related to W6. The present study indicated homology between chromosome 6M and W6 and W7 in diploid and tetraploid *Aegilops* species and a relationship with W1 and W4 in *Ae. comosa*. Chromosome 7U contains regions syntenic with W3 and W7, consistently with the previous results [21], while the 7M chromosomes were related mainly to W6 and W7 in diploid and tetraploid *Aegilops*.

Evolutionary genome rearrangements are considered to be a common phenomenon in most plant taxa, including the *Triticeae,* and to be one of the most important evolutionary driving forces for the formation of new species. Genome shuffling can be triggered by polyploidization events and is the main reason for synteny breakage in grasses since their divergence from a common ancestor. Such mosaic synteny blocks were found in rye and ryegrass when their genomes were compared with wheat [59], [57] and they were also formed during the evolution of barley and hexaploid wheat [60], [61]. Based on the comparison of orthologous regions from rice, maize, sorghum and *Brachypodium,* Murat et al. [62] proposed that chromosome shuffling events were driven by non-random centric double-strand break repair processes. The centromeric/telomeric illegitimate recombination between non-homologous chromosomes results in nested chromosome fusions, followed by additional structural changes (inversions and repeat invasions) and the formation of synteny break points [62], [63]. By investigating the hardness locus in diploid and polyploid wheat species, Chantret et al. [64] detected various genome rearrangements and suggested that illegitimate DNA recombination is one of the major evolutionary mechanisms leading to various genomic rearrangements. Recently, it became clear that retrotransposons have a definitive role in these processes [65], [66]. Genome rearrangements are also thought to induce gene duplications which lead to the pseudogenization (functionless paralogues), concerted evolution (conservation of function for paralogues), subfunctionalization (complementary function of paralogues) and neofunctionalization (novel function of paralogues) of new alleles [62]. As the investigated *Aegilops* species are closely related to *Triticum*, it can be concluded that similar mechanisms took part in the evolution of the U and M genomes. Genome rearrangements in *Aegilops*, which were also formed frequently after allopolyploid speciation [20], could have been triggered by gene duplication events, as detected in this study (Table 3). After functional divergence, these duplicated loci may serve as raw material for evolution and represent potentially useful alleles for increasing the genetic diversity of bread wheat. It should be noted that the present comparisons of *Aegilops* genomes with wheat and model species were based on 100 orthologous genes. *Ae. tauschii*, whose D genome is of similar size (5.1 pg DNA/1C) to *Ae. umbellulata* (5.05 pg DNA/1C) and *Ae. comosa* (6.18 pg DNA/1C), has approximately 36,000 genes [67], [68], so the coverage in the present work cannot be more than 0.0027×, allowing only macro level comparison. In the near future, the shotgun sequencing of individual U and M genome chromosomes isolated by flow sorting [17] will result in a much deeper comparative genome analysis of *Aegilops* (http://www.wheatgenome.org/Projects/Complimentary-Projects/Wild-Relatives) and will provide more detailed information about evolutionary rearrangements and polyploidization-related processes in *Aegilops* U and M genomes.

## Conclusions

Major efforts are underway to improve wheat yield and quality under stress conditions by increasing genetic diversity in breeding materials. Various *Aegilops* species have already been used as sources of new alleles for wheat breeding through interspecific hybridization. The conserved orthologous set markers assigned here to *Aegilops* chromosomes promise to accelerate gene introgression by facilitating the identification of alien chromatin. The analysis of complex polygenic traits such as earliness, abiotic stress tolerance and nutritional quality will also be accelerated, contributing to sustainable increases in wheat yields. Finally, the syntenic relationships between the *Aegilops* species, wheat and model species established in this work will facilitate the targeted development of new markers specific for U and M genomic regions and will contribute to the understanding of allopolyploidization-related molecular processes.

## Supporting Information

Figure S1 **Rice–wheat–** ***Aegilops*** **orthologous relationships from the genomic perspective of** ***Oryza sativa*** **.** The physical positions of the source ESTs of the COS markers are indicated on the rice chromosomes (Left). Each marker assigned to chromosomes of the wheat D genome or to chromosomes of *Ae. umbellulata* (U), *Ae. comosa* (M), *Ae. biuncialis* (U^b^, M^b^) and *Ae. geniculata* (U^g^, M^g^) is colour-coded according to the homoeologous groups of *Triticum*/*Aegilops* chromosomes. When a marker mapped to more than one wheat or *Aegilops* chromosome, other colour-coded locations are positioned adjacent to the first one. Asterisks indicate the predicted chromosomal location of a locus when the PCR amplicon was specific for the U or M genomes and could be determined unambiguously in at least one *Aegilops* species (in the diploid progenitor, or in *Ae. biuncialis* or *Ae. geniculata*) and when the highest PCR product yield in the other two species was detected in the subgenomic DNA sample containing the same chromosome.
(TIF)

Table S1 **Primer sequences and anealing temperatures of the COS markers used in the present study.**
(DOC)

Table S2 **Genomic positions of the non-polymorphic COS markers in rice and** ***Brachypodium*** **which were not assigned to** ***Aegilops*** **chromosomes.**
(DOC)

Table S3 **Results of BLASTn search of source ESTs of COS markers assigned to** ***Aegilops*** **chromosomes in the rice genomic database.**
(DOC)

Table S4 **Results of BLASTn search of source ESTs of COS markers assigned to** ***Aegilops*** **chromosomes in the** ***Brachypodium*** **genomic database.**
(DOC)

Table S5 **PCR products of the COS markers in the genotypes of wheat and** ***Aegilops*** **species.**
(DOC)

Table S6 **Assignment of COS markers to the chromosomes or to the peaks on flow karyotypes in** ***Aegilops umbellulata, Ae. comosa, Ae. biuncialis*** **and** ***Ae. geniculata*** **.**
(DOC)

Table S7 **Syntenic relationship of U and M genomes relative to wheat.**
(DOC)
