# Supplementary figures and images for: Syntenic Relationships between the U and M Genomes of Aegilops, Wheat and the Model Species Brachypodium and Rice as Revealed by COS Markers
Source: PLoS One. 2013 Aug 5;8(8):e70844. doi: 10.1371/journal.pone.0070844 (PMC3733919; doi:10.1371/journal.pone.0070844)

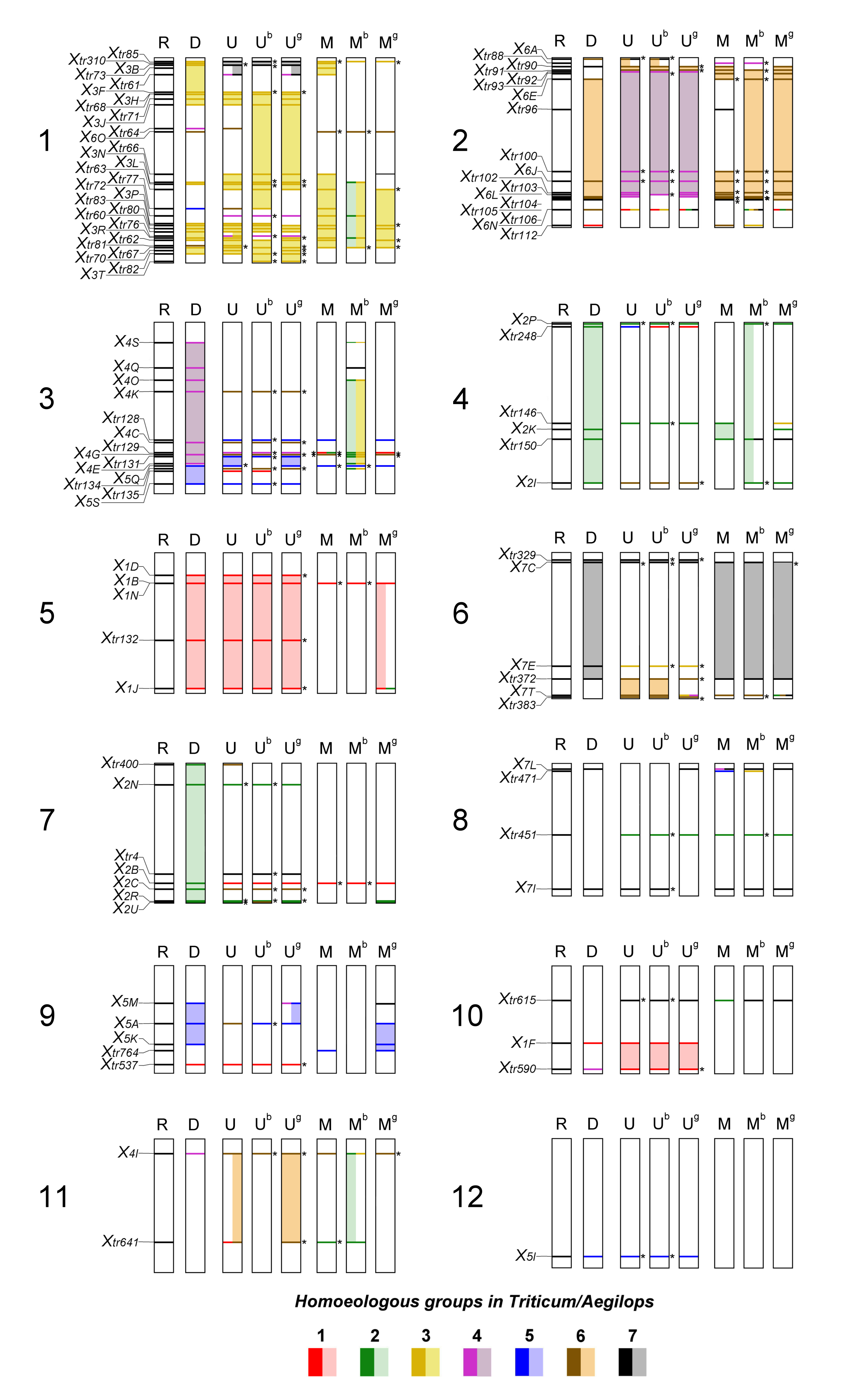

Supplement: Figure S1 — Rice–wheat– Aegilops orthologous relationships from the genomic perspective of Oryza sativa . The physical positions of the source ESTs of the COS markers are indicated on the rice chromosomes (Left). Each marker assigned to chromosomes of the wheat D genome or to chromosomes of Ae. umbellulata (U), Ae. comosa (M), Ae. biuncialis (Ub, Mb) and Ae. geniculata (Ug, Mg) is colour-coded according to the homoeologous groups of Triticum/Aegilops chromosomes. When a marker mapped to more than one wheat or Aegilops chromosome, other colour-coded locations are positioned adjacent to the first one. Asterisks indicate the predicted chromosomal location of a locus when the PCR amplicon was specific for the U or M genomes and could be determined unambiguously in at least one Aegilops species (in the diploid progenitor, or in Ae. biuncialis or Ae. geniculata) and when the highest PCR product yield in the other two species was detected in the subgenomic DNA sample containing the same chromosome. (TIF) [file pone.0070844.s001.tif]
