# Supplementary material for: Syntenic Relationships between the U and M Genomes of Aegilops, Wheat and the Model Species Brachypodium and Rice as Revealed by COS Markers
Source: PLoS One. 2013 Aug 5;8(8):e70844. doi: 10.1371/journal.pone.0070844 (PMC3733919; doi:10.1371/journal.pone.0070844)
Supplement: Table S1 — Primer sequences and anealing temperatures of the COS markers used in the present study. (DOC) [file pone.0070844.s002.doc]

**Table S1**. COS markers used in the present study together with their primer sequences and anealing temperature used to amplify products by the specific PCR programmes (WGIN or TR). Detaied information for the PCR conditions has been given in the ’Materials and Methods’.

| Marker | Forvard primer | Reverse primer | T anealing (^o^C) | PCR program |
| --- | --- | --- | --- | --- |
| *X1B* | CGCACAGAATCCAGAAATGGCACT | GATCAAAGTGTTGAGCTGTTCAGAGA | 58 | WGIN |
| *X1D* | GGCCGGTGATGAAGAAAGTGATGA | GAATCATCACTAGGGGATCCGCT | 58 | WGIN |
| *X1F* | GACTTTTGCGGGATCGAGATCCA | GTTGCTATCCCAGCAGCAGTGA | 58 | WGIN |
| *X1J* | CTTTATCATGTGCCCCTGTTGGAGA | GTCGAACTTCACTAGCCCGTACTCA | 58 | WGIN |
| *X1N* | GGATTTGATTCAAGAAGAGCCACGT | GGCTTCCCTGTATATTGTGTCAGGA | 58 | WGIN |
| *X1S* | CAAGGACAAGACCGGCAGCGT | GCCGACCACCGCGTTCTTCA | 58 | WGIN |
| *X2B* | CGAAGGCTCTGAATTCTGTCGTTCA | CTGAAAGCCCATAACTACAAGGGCA | 58 | WGIN |
| *X2C* | CTTGAAAGCGGGAGGAGTAGCTCT | GCTGGATTCCACCAAGGATCCA | 58 | WGIN |
| *X2G* | CAATGCAGACAACCAGCATCTAGT | CCCTCACCTGGAGGAAGTATGGA | 58 | WGIN |
| *X2I* | CATCAATGCTCACCACCTTCGCA | GTGGGCTTATGGATGACGCCT | 58 | WGIN |
| *X2K* | GGGTGATGTCATTGTTGCAGTGGA | CAGGAGGTCAGATTTGCCCTTGA | 58 | WGIN |
| *X2N* | CTTGATGGAAGCCATCAGCTGCA | CTGGGGCTAATGAAAGCTGAGAAGA | 58 | WGIN |
| *X2P* | TGCATGTAATCAGCGAGTCTGTCA | CCTAAGACCAACGGCCGAAGA | 58 | WGIN |
| *X2R* | CTGATGTTGACGAGAAGGTAGACGA | CGTTTCTGGTGTCGTGTCATCTTCA | 58 | WGIN |
| *X2U* | GGTGGGATAAGTTTCAAGTTGCCCT | GGAATAGGGCCATGTTGTGGTGA | 58 | WGIN |
| *X3B* | GCCAAACAGTTCCTGGTATGGAGT | GAGGCTCAACCATTGTCTTGGCT | 58 | WGIN |
| *X3F* | GGTATTGGCGAACCTGCTCTTGT | ACGATCCATGTCTCCGTCTCGA | 58 | WGIN |
| *X3H* | CCCAGCTGAGGAGGCAGTACA | GATCTACAACTACTTCCCCTACCCA | 58 | WGIN |
| *X3J* | GGCCAACAACTCCATACCACCA | GGAGAAGGAACAAGGAGTTCCCA | 58 | WGIN |
| *X3L* | CACGCTTCCTCAAGTTTCTGTGCA | CAGAAAGGTGGTGAAACCAGCACA | 58 | WGIN |
| *X3N* | GGAACCTCATTGGGTATACAACCGT | ACGTGGATAAGCTGGTTGCCCT | 58 | WGIN |
| *X3P* | TGTATGAGGCCAGGGATTCCATCA | CTTGTAGGTGCCACCAATCTTCCT | 58 | WGIN |
| *X3R* | GGTGGTACATATGCAGCTGTTCCA | TGAAGCCTACTGTGGACACTCCA | 58 | WGIN |
| *X3T* | GCTCTGAGTACAAGAGGCCTTACA | GGTCTGTCGAACACTGACTGTGA | 58 | WGIN |
| *X4A* | GGTGCGGACATGTCCATCTACTT | GGTTCTTGCCATACAGCTCAACCA | 58 | WGIN |
| *X4C* | GTCCAAGTAGAAAGCATGCTCCGA | GGCCCTGTCACAATCCTCAACT | 58 | WGIN |
| *X4E* | GGATTGATGCCCCTCTGTGATGA | ACCAGGGTGGTATTGTGGACCT | 58 | WGIN |
| *X4G* | GCAATCACGAACGGCTCGATCA | ATCTGGCAGCTTGCCAAGGCTT | 58 | WGIN |
| *X4I* | TGGATCTGCCTAGTTCGGAAGGA | TGGGAAACGGAGGAACTGGACT | 58 | WGIN |
| *X4K* | ACAAGTGTGCTGAGCATAGCGAC | CCACTGAGGCTTTGGAGAAGAAGA | 58 | WGIN |
| *X4M* | TCAGCCAGCGCTCTACTGTTCT | CTGGATGACTGGCCAACTCACAT | 58 | WGIN |
| *X4O* | GGCTGTCCAAAGGCATGTACTGA | GGTCGGAGCATATGCAGTGTACA | 58 | WGIN |
| *X4Q* | CCCTGGACAAGGAACCCTTTGA | CGAAAGACAGGGTCAGTCAGCA | 58 | WGIN |
| *X4S* | ACTGTAACATCTCCTGAGGCAGCT | CTGCTCCAACAACTGCTATTCGCA | 58 | WGIN |
| *X4U* | CAGCTTGAGTTGAAACCAGGCATG | CCTATTCCACAGCCGACATCCA | 58 | WGIN |
| *X5A* | GTGTGCTGCTCAGATGGAACCT | GTCCCTCAGCTCCAAAGCTTCA | 58 | WGIN |
| *X5C* | CATGAATGTCACCGTGAACGCGT | TTCAAGGTCTTCACGGCCTCCA | 58 | WGIN |
| *X5E* | GCGGTGTCTGAAGGATGATCTTGA | GCTGGTCCCTTATGCAGAGATACT | 58 | WGIN |
| *X5I* | CGCTGTACGAGCTCTTCAAGAAGA | TCAGCGAAGAGGCTCTCCTTCT | 58 | WGIN |
| *X5G* | GAGAGAAGCTGGGCAAAGGATGA | GGCAAGCCAAAATGATCTGGGGA | 58 | WGIN |
| *X5K* | GTATCGCCGTTTTTCCTCCTCCT | CGTACCTTCGCTTGGGAAAATGCA | 58 | WGIN |
| *X5M* | GGCACAGAACACCAGTAGTGACA | GTCCTGATTTCTTCTGGCGCTGT | 58 | WGIN |
| *X5O* | CAGCACCAGCAGGAAAATGTATGTG | GGTGACTTGCATCTGACAGGACA | 58 | WGIN |
| *X5Q* | TCTGGGTTTAGGACACCTTCCTCA | TCTCCATCCACTGGGAGGATTCA | 58 | WGIN |
| *X5S* | GCCTTGCCTTCTCATACGTACCA | GGACTGCCATTGCTCAAGGATGT | 58 | WGIN |
| *X5V* | CAGAAGGGAGAAACCTCTGCCAT | AGATGGTCTACCCTACCCGATTG | 58 | WGIN |
| *X6P* | ACCAACAGTGCCATCACCTCCA | GAGAGGTTGGCTGATCAAGGTGA | 58 | WGIN |
| *X6R* | CTTCTTGTTCCTGCTATGCAGGGA | GCAACCATCCGCGACTTAGCTA | 58 | WGIN |
| *X6A* | CAGTATACTTCGACGTTCCACTGCT | GAAGGACAACACCTACGTCTACATGT | 58 | WGIN |
| *X6C* | GAACCCAAGGGCGAGATTGACA | ACAGGGATCTCAGATGCATCTCCT | 58 | WGIN |
| *X6E* | CGTGCATCAAGAGCTTTGGAGGT | CAGAACAGCATTCTCTCGAAGAGCT | 58 | WGIN |
| *X6N* | GATTCCTACCAGCTACCAGATGCT | CGTTCCTCCTCAGTGATTCCCAA | 58 | WGIN |
| *X6O* | ACGAAGTAGTGGCTGTCGAAGGA | GCTCTTCAACTTCACGGGCAAGAA | 58 | WGIN |
| *X6J* | CAGTAGTTCTTCAGCGACTGCCA | TCAACTCCAGCCACATCAGCGA | 58 | WGIN |
| *X6L* | CTGGGCTTTACCGTGGATTCAACA | GGTCATCATCATCCTTCTGCGGA | 58 | WGIN |
| *X7A* | GCATTCAAGCATTGCTGGGAGAGA | GTACGAGCAGAAGTACCCAGAAGA | 58 | WGIN |
| *X7C* | GCTGGTTGAATCCAGGTGGTGT | CCCAAACCACTTCTTATTGGTGCT | 58 | WGIN |
| *X7E* | GGCCCATGGTAGACAGCATACA | GACAAGGCCACCAGCAACATCT | 58 | WGIN |
| *X7G* | CTCCACTGTCCACACCCGACT | CCATTCAGGAAGGGTGTTGAGCA | 58 | WGIN |
| *X7I* | CGGTGGTGCCTGTCAAGATTTACA | CGCTGCATTCCTCGACTTGATACT | 58 | WGIN |
| *X7L* | GGCTTCTCATGGAGTCAAGACACT | GATGTCCTCTGCCATTTTCTTGGCT | 58 | WGIN |
| *X7T* | ATCCTGGTCTGGAACTCGGACA | TTCGTCTGCATCCTCGCCTTCA | 58 | WGIN |
| *Xtr4* | TCAACTTCGGCGACTCCTAC | TACGCATTCAGGTAACGCAG | 62 | TR |
| *Xtr60* | AATGATGTTGGTGCTGTGGTG | ACTTCAGCCACTGATACGTTG | 62 | TR |
| *Xtr61* | AACCTGAGCCTTGTTTTGCTG | AGCACAAAGATGGAGTGAACTC | 62 | TR |
| *Xtr62* | ATGTGCTCGAAGATGCTGGAC | TGGTCTCGATGCGGAAGGTTG | 62 | TR |
| *Xtr63* | GTAGATGAGATCCTGCTTGTC | ACTTCACATGCTTGTTGTCTG | 62 | TR |
| *Xtr64* | ACTGCCGTGTATGACTGCAATG | GACTCATACCAATCGGCACAC | 62 | TR |
| *Xtr66* | ATTCCGCTGATGATGTTGAAC | CTGGAATTTCTCACCTTCTAG | 62 | TR |
| *Xtr67* | CAGAGTTTAACGTCAAAGGAC | GGCTGGTACCAGAAATCATAG | 62 | TR |
| *Xtr68* | GGACAGACAAGTTCCCTTGTC | AGCATTGATGAAACCCTTCCAG | 62 | TR |
| *Xtr70* | TTCGTATACTTCCGTGGTCTG | CTGCATATCTTCGTAGTACGTC | 62 | TR |
| *Xtr71* | GAACTCGTTGCTCACTATCTG | AATGCCACTTGTTATCCTGTG | 62 | TR |
| *Xtr72* | CGTCGTACGTCCTTGAAAAG | TGAACTCCGTGTCCATCCAGTC | 62 | TR |
| *Xtr73* | CGCTCATCAAGCAGGACTTC | GAAATCGGCGTTTCTAAGGTC | 62 | TR |
| *Xtr76* | CTATGCCGACAATCCAGAGTG | GTTGTACATCCTTTGCCAACTG | 62 | TR |
| *Xtr77* | CGATCCATTGTCTTCCAAGAG | GTTCATCCTGCCATATTCCAG | 62 | TR |
| *Xtr80* | GAGAAGCACGCCAACATCATG | TCTGAGCTCCTTGAGGTCGAC | 62 | TR |
| *Xtr81* | ATAGTTAACCTACTACTTCAGC | GTAACATCCATCCGGAATAGC | 62 | TR |
| *Xtr82* | GCTTAACACGTCATGTGCATC | GTTTGAGAATGCTTTCTGACC | 62 | TR |
| *Xtr83* | AGAGCATGCCTCTATGTCCAC | GATCTTCTTACCGATCTCGTC | 62 | TR |
| *Xtr85* | ATAACAATGGTGCCCTCGGAG | GAGTGTGCCGGAGAACCAGGAG | 62 | TR |
| *Xtr87* | AGATGTTTTCTCTGTCATGGTG | TGATAGGTCGGTACTCTGGTG | 62 | TR |
| *Xtr88* | CTTTTCTGCGCCTCCCTAAG | GTCAATAGTCTTCTTGTGGCAG | 62 | TR |
| *Xtr90* | AACAGCTCCATGAGCAACTAG | TATCTTGCATGCTCCTCTATG | 62 | TR |
| *Xtr91* | GCGAAAGCGCAAATCGTTCTC | AAAACGATCACGAAGAGGATG | 62 | TR |
| *Xtr92* | TTGAATCGCACGCCTTCGGAC | CATGTCATAGCAGGAAGCTTG | 62 | TR |
| *Xtr93* | TTCAATTGGTCCAGTAGAAGG | GTGTCTGTGTAGCTCCTC | 62 | TR |
| *Xtr94* | ACAATGATCTCCTCACGTCTC | GTGGTTGCAGATATTTCTTTCTC | 62 | TR |
| *Xtr96* | TTTCACCCGGATACACCCATG | CTCGCAGGTCATATAACCTG | 62 | TR |
| *Xtr97* | GTTCTGGTCATCCTTGATGAC | TCGCATGCACAACTCCTCAC | 62 | TR |
| *Xtr99* | GTGCAGCAGTAGGGACAGATG | CAGACATATCCGAGATTGAAG | 62 | TR |
| *Xtr100* | CATATTCCACCGATTCTTCTC | ATGTGGAAACTGTTGGCAAAC | 62 | TR |
| *Xtr101* | GGCTAGATGATTCCGGAGGCT | CAAGTGCTGATTGTAGAAGTG | 62 | TR |
| *Xtr102* | ATGGCCCAGACACTTACTTTG | GATAAGCTCATCTAGTCTGTC | 62 | TR |
| *Xtr103* | CGGCACGCCCAAGGACAC | CTTCGCCAATTGCAGGAC | 62 | TR |
| *Xtr104* | TTGGTCTAGACCATCCTG | TTCGGTTTGATTGAGCCCATG | 62 | TR |
| *Xtr105* | TTTCTACTGCCAGAGGACATC | GCTGTAAGAGTTAGGATCATC | 62 | TR |
| *Xtr106* | AACATGATATCCCCTGTGCAC | AATGATGCTACTTTCTCGCTG | 62 | TR |
| *Xtr107* | CAAGAAGTGGTATTACTACAG | TTGACCTCAATCAGAGCTCAC | 62 | TR |
| *Xtr108* | TGCCGCTTATTTTTGACCGTG | TCTTGGGTAAGTTGGAAATGC | 62 | TR |
| *Xtr110* | GTCAAATCTCCAACTTTCCAG | TGAGTGGAAAGCTCTAAGCAC | 62 | TR |
| *Xtr112* | CCATTGAAAGGAGGATAGCAG | TTCATAGTGCAGTACTTGGAG | 62 | TR |
| *Xtr126* | TTGATGTTCTGTCCGGGTTAC | GACAGAAGATATGTCACTGTC | 62 | TR |
| *Xtr128* | TCTGAAGAAACCTGGAGGATC | ATTTGCTTTGGGTTGACCAAG | 62 | TR |
| *Xtr129* | CAGGAAGTTGGTACCATTGTG | GTCAAGCGTGAGGATTTTAAG | 62 | TR |
| *Xtr131* | AAAGACCACCTCACCACCGTC | AGTACTGGTCACAGATGTAAC | 62 | TR |
| *Xtr134* | TTGCTTGGTTCCTCCTTGGTG | AGGAGCTGACCCAAATAGCTG | 62 | TR |
| *Xtr135* | TCTCGACCATTATCTTTCCTG | AATTGGAAGTTGAATTGCTGTC | 62 | TR |
| *Xtr143* | CCAGAGAGATCTTGAAGTCATC | TTCAGCCAGTCTTCTCTGCAG | 62 | TR |
| *Xtr146* | GGACAGGTTCTACAGGCAGAG | CTCTGCGGATTTGGTACTTTC | 62 | TR |
| *Xtr150* | ATCTCTCGGAGAAAGACAGAG | GATCAAATTTCTCTGGCGTCAG | 62 | TR |
| *Xtr154* | CTGTGTTGATGGTGATGGATC | ATGCTGGACTATGCGAAACAC | 62 | TR |
| *Xtr170* | ATTCCTGTTGATGAGCGGAT | GCTCAACGACTGAGATTCCC | 62 | TR |
| *Xtr232* | ATCTGGAGACAAACGGGATG | GAAGAATTATTCGAGGGGGC | 62 | TR |
| *Xtr248* | GTCTCCTCCGACGTGCTC | GAAGTTACGGCCAAGGATCA | 62 | TR |
| *Xtr310* | TGGGGCATACTTGAGAAGAA | GGAGCTTGTGGAAACTCAGG | 62 | TR |
| *Xtr329* | AAGAGCTGTGGTTGTTGGCT | CCTCATTCTTGGTACGCCAT | 62 | TR |
| *Xtr330* | GAAATGAAGGAGTCCACGCT | CAGCAACCAGATTCAGCAAA | 62 | TR |
| *Xtr366* | TGCTGGCCAGACTGATCTAA | AAGAATGCTGCAAATGGACC | 62 | TR |
| *Xtr372* | CAGTGGTGGCTGCTCTTTTT | TCCCCAGATGCTAGTGGAAG | 62 | TR |
| *Xtr383* | TGACATCTTCGTCTGCATCC | GACCATCTCAACGACAGGCT | 62 | TR |
| *Xtr393* | GCGTTAGCCACTGCATTACA | AACCATCAAAGAGCTTCCCA | 62 | TR |
| *Xtr400* | GAAGAGAGAAGCTGAAGCGG | GCGGGAGTGACACTGTTTTT | 62 | TR |
| *Xtr413* | GGTGTTCCACTCACCGATCT | TCCATCACGAAGCAACCATA | 62 | TR |
| *Xtr437* | AGGTCGACATCTCCACGG | TCCCCAGTCTCCTTGATGAC | 62 | TR |
| *Xtr451* | GATGAGAAGCACATTGGGGT | CATTGATCCTCCTCTGCCAT | 62 | TR |
| *Xtr462* | GTGGAGACCCCAGCTCAAG | CGCCACCTCAAAGATCTCAT | 62 | TR |
| *Xtr471* | AGCCCAGGAGTCTCTTGACA | CTGCTCTTTTGTCCTACCGC | 62 | TR |
| *Xtr488* | TTTTTGCGAAGAAGGTACAAATC | TGACCTTTGCTTGAACTCCC | 62 | TR |
| *Xtr537* | CTTCGACTTGTCGCTGAACA | GTAGCTACGCCACTCGCTCT | 62 | TR |
| *Xtr570* | GGAGAACCGTGTGGTTCAGT | AAACATATGCTGCTTTGGGC | 62 | TR |
| *Xtr590* | GTGCCCTTCATCGTCCTG | AGTATCCCTTGGATCCCGAC | 62 | TR |
| *Xtr615* | AATATTTGGTCTCATCACTTACTTCA | GGAGGTAAAGGAGCAATTTCC | 62 | TR |
| *Xtr641* | GCAGCATTTAGAGCAACACTG | ACACAACCAACTTCGGAACC | 62 | TR |
| *Xtr654* | GAATATTGGGAGACACTATACTCATCA | ACATGTGGAGAACTGTGCCA | 62 | TR |
| *Xtr725* | GTCCTTCATCTTCGGGGACT | CGTAGTTCACGCCGTTCAG | 62 | TR |
| *Xtr731* | AGAAAGGAAAAGGGTGGCAT | AGGTTCCATTCCAGATGTGC | 62 | TR |
| *Xtr757* | CATGACGGTGGAGAAGTTCA | AGAAGGATGAGCCTCAGCAA | 62 | TR |
| *Xtr764* | CCGACTCCAAGAAGAGGAGA | CTTTATCTTTGCTCTGGGCG | 62 | TR |
