# Supplementary material for: Syntenic Relationships between the U and M Genomes of Aegilops, Wheat and the Model Species Brachypodium and Rice as Revealed by COS Markers
Source: PLoS One. 2013 Aug 5;8(8):e70844. doi: 10.1371/journal.pone.0070844 (PMC3733919; doi:10.1371/journal.pone.0070844)
Supplement: Table S2 — Genomic positions of the non-polymorphic COS markers in rice and Brachypodium which were not assigned to Aegilops chromosomes. (DOC) [file pone.0070844.s003.doc]

**Table S2.** Rice and *Brachypodium* genomic positions of the source sequences of COS markers amplified nonpolymorphic PCR products between wheat and *Aegilops* genotypes and not assigned to *Aegilops* chromosomes. The source sequences (shown as Accession No.) of the markers were found in the The Institute of Genomic Research (TIGR) database (<http://plantta.jcvi.org/index.shtml>) and used as queries for BLASTn search using ‛megablast’ as default parameter in the Ensembl Plants Database (<http://plants.ensembl.org/>. The start positions of the best hits, characterized by their BLAST parameters (E-value, % of Identity and Alignment length).

| Marker | Source sequence | Best hit in rice | | Best hit in *Brachypodium* | |
| --- | --- | --- | --- | --- | --- |
|  |  | BLAST parameters  (E value/ID%/ Alignment length) | Location  (chromosome, position in bp) | BLAST parameters  (E value/ID%/ Alignment length) | Location  (chromosome, position in bp) |
| *X1S* | BE590719 | 1.0e-33/69.50/400 | Chr05;  26,865,216 | 1.8e-32/71.97/421 | Chr02;  16064115 |
| *X2G* | BE442608 | 2.1e-78/88.35/206 | Chr07;  801,139 | 7.8e-131/90.10/202 | Chr01;  58116642 |
| *X4A* | BE498428 | 1.7e-204/88.44/225 | Chr03;  16,302,593 | 6.5e-194/91.30/230 | Chr01;  59637303 |
| *X4M* | BE443253 | 7.9e-73/87.04/162 | Chr03;  11,396,811 | 6.5e-96/80.08/251 | Chr01;  63297730 |
| *X4U* | BE444334 | 5.1e-70/88.15/211 | Chr07;  5,751,730 | 3.4e-104/91.51/212 | Chr01;  52699973 |
| *X5C* | BF292081 | 1.3e-21/79.90/199 | Chr12;  25,953,678 | 4.3e-29/78.42/241 | Chr04;  1,160,973 |
| *X5E* | BE496976 | 3.0e-118/88.14/177 | Chr12;  19,425,037 | 1.7e-171/87.15/638 | Chr02;  23,900,021 |
| *X5G* | BE500291 | 2.2e-48/85.44/103 | Chr11;  24,739,512 | 1.1e-151/82.75/400 | Chr04,  5,298,920 |
| *X5V* | BE403214 | 1.7e-62/83.82/346 | Chr03;  35,253,348 | 1.6e-77/92.40/329 | Chr01,  1,631,662 |
| *X6P* | BE495143 | 1.3e-36/75.98/204 | Chr02;  33,476,004 | 7.3e-55/73.93/280 | Chr03,  54,307,429 |
| *X6R* | BG313802 | 2.2e-201/87.61/339 | Chr02;  31,759,043 | 1.1e-213/84.46/592 | Chr03,  58,139,161 |
| *X7A* | BF202702 | 0/85.61/1147 | Chr06;  1,808,026 | 0/90.66/1017 | Chr01,  50,093,331 |
| *Xtr94* | CA666063 | 2.8e-48/81.76/318 | Chr02;  4,632,261 | 2.2e-55/91.76/279 | Chr03,  4,457,432 |
| Xtr97 | TA18623_4565 | 2.7e-215/86.07/438 | Chr02;  15,927,366 | 1.6e-246/86.04/480 | Chr03,  44,762,733 |
| Xtr99 | TA24826_4565 | 1.5e-117/84.58 /577 | Chr02;  23,264,284 | 7.8e-146/89.46/607 | Chr03,  49,439,148 |
| Xtr101 | TA29417_4565 | 2.9e-93/88.55/332 | Chr02;  26,155,147 | 1.8e-131/93.07/332 | Chr03,  51,467,810 |
| Xtr110 | CD876986 | 7.9e-48/82.48 /234 | Chr02;  33,997,188 | 1.0e-68/80.78/255 | Chr03,  54,780,023 |
| Xtr126 | TA20698_4565 | 1.0e-231/90.86/700 | Chr03;  3,986,811 | 2.4e-229/92.34/705 | Chr01,  70,578,451 |
| Xtr143 | TA33419_4565 | 2.0e-34/84.73/262 | Chr04;  16,369,100 | 5.2e-74/80.57/314 | Chr05,  6,871,042 |
| Xtr154 | TA34832_4565 | 4.7e-59/71.59/630 | Chr04;  31,033,743 | 7.0e-49/68.72/633 | Chr05,  24,301,585 |
| Xtr330 | TA71281_4565 | 6.8e-118/82.21/163 | Chr06;  14,504,939 | 1.2e-166/85.90/227 | Chr03,  21,843,083 |
| Xtr366 | TA89976_4565 | 1.4e-165/87.22/313 | Chr06;  29,770,166 | 1.6e-167/88.10/395 | Chr01;  30,109,270 |
| Xtr413 | TA89504_4565 | 1.0e-97/84.84/310 | Chr07;  7,153,507 | 3.6e-115/93.01/272 | Chr02;  37,355,032 |
| Xtr462 | TA57781_4565 | 2.9e-109/88.89/153 | Chr08;  9,072,865 | 5.2e-118/79.16/499 | Chr03;  17,597,556 |
| Xtr488 | TA83063_4565 | 4.2e-109/81.15/382 | Chr04;  25,839,866 | 1.3e-134/84.92/431 | Chr05;  19,361,924 |
| Xtr570 | TA67264_4565 | 0/87.00/808 | Chr09;  14,412,803 | 0/91.58/808 | Chr01;  50,356,217 |
| Xtr654 | TA97094_4565 | 4.7e-73/74.54/538 | Chr11;  23,429,316 | 2.1e-77/83.58/402 | Chr04;  14,088,230 |
| Xtr731 | TA68676_4565 | 1.8e-50/85.44/309 | Chr07;  26,019,453 | 1.3e-100/87.99/308 | Chr01;  16,492,098 |
| Xtr757 | TA84129_4565 | 2.0e-86/82.51/223 | Chr09;  21,227,691 | 2.3e-103/79.12/297 | Chr04;  41,720,472 |
