# Supplementary material for: Syntenic Relationships between the U and M Genomes of Aegilops, Wheat and the Model Species Brachypodium and Rice as Revealed by COS Markers
Source: PLoS One. 2013 Aug 5;8(8):e70844. doi: 10.1371/journal.pone.0070844 (PMC3733919; doi:10.1371/journal.pone.0070844)
Supplement: Table S3 — Results of BLASTn search of source ESTs of COS markers assigned to Aegilops chromosomes in the rice genomic database. (DOC) [file pone.0070844.s004.doc]

**Table S3.** Results of BLASTn search for COS markers assigned to *Aegilops* chromosomes in the rice genomic database. The source sequences (shown as Accession No.) of the markers were found in the The Institute of Genomic Research (TIGR) database (<http://plantta.jcvi.org/index.shtml>) and used as queries for BLASTn search using ‛megablast’ as default parameter in the Ensembl Plants Database (<http://plants.ensembl.org/>. The start positions of the best hits, characterized by their BLAST parameters (E-value, % of Identity and Alignment length), were used to construct physical maps of the COS markers in rice.

| Marker | Source sequence | Best hit | |
| --- | --- | --- | --- |
|  |  | BLAST parameters  (E value/ID%/ Alignment length) | Location  (chromosome, position in bp) |
| *X1B* | BG262247 | 7.2e-129/80.77/104 | Chr05;  6,699,137 |
| *X1D* | BG606097 | 1.8e-94/62.59/278 | Chr05;  4,969,604 |
| *X1F* | BE403420 | 2.6e-75/67.94/315 | Chr10;  16,707,340 |
| *X1J* | BE398263 | 2.7e-90/62.65/332 | Chr05;  28,811,844 |
| *X1N* | BG262247 | 2.1e-131/85.91/369 | Chr05;  6,699,137 |
| *X2B* | BE496863 | 1.9e-28/69.47/321 | Chr07;  25,430,474 |
| *X2C* | BF201328 | 2.9e-23/81.98/111 | Chr07;  26,736,951 |
| *X2I* | BE444894 | 5.6e-98/81.80/467 | Chr04;  33,988,889 |
| *X2K* | BE426364 | 4.8e-65/74.60/374 | Chr04;  22,772,533 |
| *X2N* | BE444851 | 8.4e-48/71.03/214 | Chr07;  4,655,972 |
| *X2P* | BE444541 | 6.9e-34 / 84.67/261 | Chr04;  0,397,790 |
| *X2R* | BG275030 | 3.8e-29/81.36/118 | Chr07;  29,543,956 |
| *X2U* | BG275030 | 8.8e-08/62.78/266 | Chr07;  29,542,888 |
| *X3B* | BE438292 | 8.1e-21/72.44/225 | Chr01;  1,601,137 |
| *X3F* | BE488644 | 3.1e-22/71.82/220 | Chr01;  7,556,655 |
| *X3H* | BE488921 | 1.2e-13/60.29/544 | Chr01;  7,719,147 |
| *X3J* | BF483259 | 3.8e-41/76.67/270 | Chr01;  10,006,147 |
| *X3L* | BE494776 | 1.7e-48/69.30/228 | Chr01;  26,534,079 |
| *X3N* | BG263667 | 2.9e-16/62.10/219 | Chr01;  26,526,512 |
| *X3P* | BF478406 | 5.5e-42/77.92/231 | Chr01;  35,175,052 |

| *X3R* | BF145691 | 9.2e-45/95.65/184 | Chr01;  37,855,586 |
| --- | --- | --- | --- |
| *X3T* | BF200774 | 1.2e-44/74.16/209 | Chr01;  42,976,677 |
| *X4C* | BF484620 | 9.3e-31/79.85/273 | Chr03;  25,590,654 |
| *X4E* | BE498699 | 2.2e-44/86.15/195 | Chr03;  30,176,187 |
| *X4G* | BE442666 | 4.9e-59/70.77 / 366 | Chr03;  28,086,005 |
| *X4I* | BG275006 | 1.1e-41/87.88/165 | Chr11;  3,288,174 |
| *X4K* | BF202969 | 1.6e-20/78.85/156 | Chr03;  14,850,098 |
| *X4O* | BE426016 | 1.1e-34/74.29/210 | Chr03;  12,405,109 |
| *X4Q* | BE497618 | 7.2e-80/85.59/222 | Chr03;  9,842,554 |
| *X4S* | BF485078 | 7.7e-27/71.35/185 | Chr03;  4,497,183 |
| *X5A* | BE399966 | 2.8e-08/74.6/126 | Chr09;  12,541,209 |
| *X5I* | BE352603 | 6.4e-29/80.98/163 | Chr12;  25,033,273 |
| *X5K* | BE443187 | 6.6e-60/79.38/388 | Chr09;  16,854,796 |
| *X5M* | BE499599 | 1.8e-57/84.39/205 | Chr09;  8,151,670 |
| *X5Q* | BF483771 | 7.4e-66/90.26/380 | Chr03;  30,526,184 |
| *X5S* | BE406609 | 1.1e-90/80.88/340 | Chr03;  34,328,406 |
| *X6A* | BE496826 | 2.0e-31/88.78/196 | Chr02;  0,184,258 |
| *X6E* | BE446153 | 5.2e-19/79.02/205 | Chr02;  4,719,823 |
| *X6J* | BE591696 | 7.0e-32/79.08/196 | Chr02;  26,151,165 |
| *X6L* | BE426214 | 1.6e-141/86.56/424 | Chr02;  29,818,195 |
| *X6N* | BE490226 | 1.8e-58/83.26/233 | Chr02;  32,062,092 |
| *X6O* | BF202810 | 1.6e-153/86.32/380 | Chr01;  15,674,233 |
| *X7C* | BE518351 | 5.9e-47/80.09/226 | Chr06;  2,248,470 |
| *X7E* | BE497999 | 4.8e-86/92.59/297 | Chr06;  24,403,717 |
| *X7I* | BE404728 | 4.2e-40/82.16/269 | Chr08;  26,918,046 |
| *X7L* | BE498418 | 7.7e-30/74.75/198 | Chr08;  1,668,402 |
| *X7T* | BF484041 | 7.7e-56/87.85/354 | Chr06;  31,033,873 |
| *Xtr4* | CK206721 | 1.1e-91/77.03/296 | Chr07;  23,813,751 |

| *Xtr60* | CA617186 | 2.4e-48/82.52/326 | Chr01;  36,670,439 |
| --- | --- | --- | --- |
| *Xtr61* | CA641245 | 2.5e-27/82.59/201 | Chr01;  3,706,935 |
| *Xtr62* | CD454313 | 4.6e-40/73.77/305 | Chr01;  38,478,808 |
| *Xtr63* | CK214036 | 4.0e-75/82.81/384 | Chr01;  27,744,867 |
| *Xtr64* | CV776062 | 1.2e-44/84.02/194 | Chr01;  15,021,808 |
| *Xtr66* | TA12687_4565 | 0/100.0/156425 | Chr01;  26,080,352 |
| *Xtr67* | TA1389_4565 | 0/100.0/138136 | Chr01;  41,581,881 |
| *Xtr68* | TA1394_4565 | 3.4e-128/100.0/100 | Chr01;  7,721,188 |
| *Xtr70* | TA15755_4565 | 8.0e-211/100.0/171 | Chr01;  40,632,868 |
| *Xtr71* | TA19207_4565 | 1.7e-58/77.07/423 | Chr01;  8,794,211 |
| *Xtr72* | TA22305_4565 | 5.6e-105/100.0/125 | Chr01;  33,408,669 |
| *Xtr73* | TA22494_4565 | 1.5e-58/77.87/122 | Chr01;  2,383,306 |
| *Xtr76* | TA24322_4565 | 0/100.0/178830 | Chr01;  39,302,115 |
| *Xtr77* | TA28162_4565 | 0/100.0/130 | Chr01;  31,859,491 |
| *Xtr80* | TA36258_4565 | 0/100.0/170759 | Chr01;  37,498,131 |
| *Xtr81* | TA37064_4565 | 2.0e-81/74.21/221 | Chr01;  39,702,650 |
| *Xtr82* | TA380_4565 | 0/100.0/130355 | Chr01;  42,985,574 |
| *Xtr83* | TA41313_4565 | 0/100.0/308 | Chr01;  35,256,984 |
| *Xtr85* | TA48966_4565 | 0/100.0/78600 | Chr01;  1,145,698 |
| *Xtr88* | CK215999 | 0/100.0/147735 | Chr02;  1,318,729 |
| *Xtr90* | TA47790_4565 | 6.0e-199/100.0/217 | Chr02;  2,078,132 |
| *Xtr91* | TA30863_4565 | 0/100.0/161081 | Chr02;  2,874,362 |
| *Xtr92* | BQ239241 | 0/100.0/487 | Chr02;  3,214,835 |
| *Xtr93* | CK155919 | 0/100.0/122214 | Chr02;  3,387,178 |
| *Xtr96* | TA37542_4565 | 4.9e-31/84.21/114 | Chr02;  11,059,257 |
| *Xtr100* | CD866256 | 1.9e-93/86.95/383 | Chr02;  24,087,208 |
| *Xtr102* | CD491457 | 8.8e-188/100.0/182 | Chr02;  28,696,146 |
| *Xtr103* | TA21621_4565 | 0/100.0/114511 | Chr02;  29,785,311 |

| *Xtr104* | TA15448_4565 | 0/99.38/161 | Chr02;  29,758,505 |
| --- | --- | --- | --- |
| *Xtr105* | DR739950 | 0/100.0/123428 | Chr02;  30,612,024 |
| *Xtr106* | TA19311_4565 | 0/98.39/310 | Chr02;  29,859,256 |
| *Xtr112* | TA37912_4565 | 0/100.0/171421 | Chr02;  36,351,977 |
| *Xtr128* | TA18489_4565 | 0/100.0/129900 | Chr03;  25,811,480 |
| *Xtr129* | CD865921 | 0/100.0/119329 | Chr03;  28,865,682 |
| *Xtr131* | TA20131_4565 | 3.6e-177/100.0/124 | Chr03;  28,463,889 |
| *Xtr134* | TA11490_4565 | 8.8e-116/92.81/167 | Chr03;  31,118,496 |
| *Xtr135* | DR738865 | 0/100.0/159091 | Chr03;  32,650,418 |
| *Xtr146* | CA635158 | 4.5e-91/99.19/123 | Chr04;  21,457,616 |
| *Xtr150* | TA8222_4565 | 0/99.72/352 | Chr04;  24,761,519 |
| *Xtr248* | TA346_4565 | 2.5e-89/85.27/292 | Chr05;  18,783,682 |
| *Xtr232* | TA21859_4565 | 4.6e-190/88.55/795 | Chr04;  1,132,675 |
| *Xtr310* | TA22013_4565 | 1.6e-224/81.56/450 | Chr01;  1,424,545 |
| *Xtr329* | TA61249_4565 | 4.7e-121/86.43/258 | Chr06;  1,816,293 |
| *Xtr372* | TA82503_4565 | 3.7e-55/74.71/257 | Chr06;  27,087,629 |
| *Xtr383* | TA76259_4565 | 1.8e-62/74.48/388 | Chr06;  31,032,641 |
| *Xtr400* | TA69542_4565 | 8.7e-176/81.73/542 | Chr07;  39,449 |
| *Xtr451* | TA81179_4565 | 8.5e-96/76.67/390 | Chr08;  15,394,279 |
| *Xtr471* | BG910093 | 3.4e-72/86.43/221 | Chr08;  1,712,618 |
| *Xtr537* | TA71452_4565 | 1.9e-82/82.25/276 | Chr09;  21,155,756 |
| *Xtr590* | TA72902_4565 | 8.8e-55/76.50/200 | Chr10;  22,149,393 |
| *Xtr615* | CJ552319 | 1.6e-64/78.75/320 | Chr10;  7,534,387 |
| *Xtr641* | TA109074_4565 | 7.8e-47/65.06/312 | Chr11;  22,094,137 |
| *Xtr764* | TA95943_4565 | 3.2e-66/80.0/235 | Chr09;  18,141,437 |
