# Supplementary material for: Syntenic Relationships between the U and M Genomes of Aegilops, Wheat and the Model Species Brachypodium and Rice as Revealed by COS Markers
Source: PLoS One. 2013 Aug 5;8(8):e70844. doi: 10.1371/journal.pone.0070844 (PMC3733919; doi:10.1371/journal.pone.0070844)
Supplement: Table S4 — Results of BLASTn search of source ESTs of COS markers assigned to Aegilops chromosomes in the Brachypodium genomic database. (DOC) [file pone.0070844.s005.doc]

**Table S4.** Results of BLASTn search for COS markers assigned to *Aegilops* chromosomes in the *Brachypodium* genomic database. The source sequences (shown as Accession No.) of the markers were found in The Institute of Genomic Research (TIGR) database (<http://plantta.jcvi.org/index.shtml>) and used as queries for BLASTn search using ‛megablast’ as default parameter in the Ensembl Plants Database (<http://plants.ensembl.org/>. The start positions of the best hits, characterized by their BLAST parameters (E-value, % of Identity and Alignment length), were used to construct physical maps of the COS markers in *Brachypodium*.

| Marker | Source sequence | Best hit | |
| --- | --- | --- | --- |
|  |  | BLAST parameters  (E value / ID% / Alignment length) | Location  (chromosome, position in bp) |
| *X1B* | BG262247 | 1.2e-155/91.83/367 | Chr02,  32,518,246 |
| *X1D* | BG606097 | 4.5e-117/80.13/302 | Chr02,  33,714,933 |
| *X1F* | BE403420 | 5.2e-97/78.22/202 | Chr03,  29,194,845 |
| *X1J* | BE398263 | 4.0e-131/93.75/176 | Chr02,  13,429,745 |
| *X1N* | BG262247 | 1.2e-155/91.83/367 | Chr02,  32,518,246 |
| *X2B* | BE496863 | 9.5e-70/90.91/154 | Chr01,  45,217,855 |
| *X2C* | BF201328 | 7.2e-54/ 93.59/156 | Chr01,  21,485,201 |
| *X2I* | BE444894 | 1.2e-190/86.48/488 | Chr05,  26,858,844 |
| *X2K* | BE426364 | 6.4e-118/93.59/234 | Chr05,  15,845,264 |
| *X2N* | BE444851 | 2.0e-61/84.46/148 | Chr01,  53,402,580 |
| *X2P* | BE444541 | 1.4e-98/81.30/385 | Chr05,  2,252,061 |
| *X2R* | BG275030 | 1.9e-46/85.71/126 | Chr01,  13,396,899 |
| *X2U* | BG275030 | 1.9e-46/85.71/126 | Chr01,  13,396,899 |
| *X3B* | BE438292 | 1.5e-188/84.57/473 | Chr02,  1,355,457 |
| *X3F* | BE488644 | 1.2e-45/82.59/247 | Chr02,  6,538,170 |
| *X3H* | BE488921 | 5.6e-84/93.55/124 | Chr02,  6,723,870 |
| *X3J* | BF483259 | 2.3e-63/82.37/278 | Chr02,  9,096,524 |
| *X3L* | BE494776 | 2.3e-49/77.65/179 | Chr02,  45,788,448 |
| *X3N* | BG263667 | 2.3e-95/92.02/213 | Chr01,  72,974,055 |
| *X3P* | BF478406 | 2.6e-62/78.02/323 | Chr02,  52,751,637 |

| *X3R* | BF145691 | 2.8e-57/89.42/208 | Chr02,  54,798,080 |
| --- | --- | --- | --- |
| *X3T* | BF200774 | 4.2e-87/80.38/265 | Chr02,  58,982,906 |
| *X4C* | BF484620 | 1.5e-97/78.13/654 | Chr01,  10,067,029 |
| *X4E* | BE498699 | 1.7e-162/91.82/428 | Chr01,  6,795,167 |
| *X4G* | BE442666 | 2.5e-228/82.99/335 | Chr01,  8,669,373 |
| *X4I* | BG275006 | 8.4e-63/73.03/267 | Chr04,  29,884,602 |
| *X4K* | BF202969 | 2.5e-61/83.27/245 | Chr01,  60,661,344 |
| *X4O* | BE426016 | 7.4e-69/74.08/463 | Chr01,  62,412,522 |
| *X4Q* | BE497618 | 6.5e-38/57.14/350 | Chr01,  68,062,460 |
| *X4S* | BF485078 | 4.2e-28/70.67/208 | Chr01,  69,980,179 |
| *X5A* | BE399966 | 3.5e-10/76.98/126 | Chr04,  34,687,522 |
| *X5I* | BE352603 | 1.4e-63/80.39/153 | Chr04,  2,034,953 |
| *X5K* | BE443187 | 6.3e-115/79.87/457 | Chr04,  37,559,944 |
| *X5M* | BE499599 | 9.4e-88/76.72/378 | Chr04,  33,071,173 |
| *X5Q* | BF483771 | 1.3e-224/92.75/414 | Chr01,  6,387,888 |
| *X5S* | BE406609 | 1.3e-97/88.39/310 | Chr01,  2,631,038 |
| *X6A* | BE496826 | 1.0e-123/91.94/248 | Chr03,  0,344,412 |
| *X6E* | BE446153 | 4.5e-61/76.49/285 | Chr03,  4,506,323 |
| *X6J* | BE591696 | 2.5e-129/87.29/181 | Chr03,  51,463,478 |
| *X6L* | BE426214 | 1.9e-97 /82.07/736 | Chr03,  54,189,866 |
| *X6N* | BE490226 | 2.1e-142/89.82/275 | Chr03,  57,704,456 |
| *X6O* | BF202810 | 3.7e-142/78.42/482 | Chr02,  11,623,838 |
| *X7C* | BE518351 | 9.2e-151/74.51/357 | Chr01,  49,558,517 |
| *X7E* | BE497999 | 0/92.67/1051 | Chr02,  48,354,825 |
| *X7I* | BE404728 | 2.1e-114/89.17/277 | Chr03,  43,508,959 |
| *X7L* | BE498418 | 3.4e-173/76.35/537 | Chr03,  12,697,062 |
| *X7T* | BF484041 | 2.0e-206/91.11/720 | Chr01,  25,174,226 |
| *Xtr4* | CK206721 | 1.7e-88 /79.18/293 | Chr01,  18,552,967 |

| *Xtr60* | CA617186 | 8.3e-52/93.38/272 | Chr02,  53,827,758 |
| --- | --- | --- | --- |
| *Xtr61* | CA641245 | 2.1e-29/76.34/317 | Chr02,  3,132,712 |
| *Xtr62* | CD454313 | 3.4e-71/71.16/371 | Chr02,  55,406,986 |
| *Xtr63* | CK214036 | 2.4e-75/81.36/338 | Chr02,  46,834,070 |
| *Xtr64* | CV776062 | 1.8e-54/84.48/232 | Chr02,  11,325,241 |
| *Xtr66* | TA12687_4565 | 1.3e-294/87.79/262 | Chr02,  44,113,048 |
| *Xtr67* | TA1389_4565 | 1.5e-138/91.32/288 | Chr02,  56,492,855 |
| *Xtr68* | TA1394_4565 | 4.2e-38/93.55/124 | Chr02,  6,723,870 |
| *Xtr70* | TA15755_4565 | 7.9e-194/82.52/795 | Chr02,  57,116,295 |
| *Xtr71* | TA19207_4565 | 5.1e-159/94.79/288 | Chr02,  7,797,772 |
| *Xtr72* | TA22305_4565 | 9.1e-75/87.47/375 | Chr02,  51,610,753 |
| *Xtr73* | TA22494_4565 | 3.1e-47/78.71/202 | Chr02,  2,135,426 |
| *Xtr76* | TA24322_4565 | 2.7e-59/90.00/150 | Chr02,  54,633,255 |
| *Xtr77* | TA28162_4565 | 5.7e-225/91.71/712 | Chr02,  50,281,129 |
| *Xtr80* | TA36258_4565 | 3.7e-39/67.39/506 | Chr02,  53,345,639 |
| *Xtr81* | TA37064_4565 | 3.6e-98/89.84/305 | Chr02,  56,281,811 |
| *Xtr82* | TA380_4565 | 2.7e-59/87.08/240 | Chr02,  57,741,407 |
| *Xtr83* | TA41313_4565 | 3.2e-77/82.96/311 | Chr02,  52,805,290 |
| *Xtr85* | TA48966_4565 | 3.4e-76/79.15/259 | Chr02,  1,073,931 |
| *Xtr88* | CK215999 | 6.9e-40/70.45/308 | Chr03,  1,548,602 |
| *Xtr90* | TA47790_4565 | 2.3e-13/58.44/486 | Chr03,  2,322,505 |
| *Xtr91* | TA30863_4565 | 2.0e-50/88.70/230 | Chr03,  2,838,135 |
| *Xtr92* | BQ239241 | 5.7e-44/75.00/184 | Chr03,  3,075,394 |
| *Xtr93* | CK155919 | 2.8e-44/69.85/262 | Chr03,  3,349,656 |
| *Xtr96* | TA37542_4565 | 8.9e-51/80.00/170 | Chr03,  8,836,975 |
| *Xtr100* | CD866256 | 1.9e-102/91.37 /394 | Chr03,  49,951,727 |
| *Xtr102* | CD491457 | 2.8e-66/77.75/391 | Chr03,  53,307,612 |
| *Xtr103* | TA21621_4565 | 1.6e-104/88.71/629 | Chr03,  54,634,403 |

| *Xtr104* | TA15448_4565 | 2.5e-93/89.66/348 | Chr03,  54,108,006 |
| --- | --- | --- | --- |
| *Xtr105* | DR739950 | 2.8e-131/88.22/501 | Chr03,  54,203,630 |
| *Xtr106* | TA19311_4565 | 0/87.28/566 | Chr01,  50,634,262 |
| *Xtr112* | TA37912_4565 | 2.1e-58/73.93/257 | Chr03,  56,522,655 |
| *Xtr128* | TA18489_4565 | 8.2e-95/91.00/489 | Chr02,  14,532,155 |
| *Xtr129* | CD865921 | 5.3e-64/79.26/270 | Chr01,  8,705,154 |
| *Xtr131* | TA20131_4565 | 1.1e-116/87.30/488 | Chr01,  72,772,576 |
| *Xtr134* | TA11490_4565 | 1.5e-229/85.43/508 | Chr01,  5,733,980 |
| *Xtr135* | DR738865 | 1.0e-83/80.37/214 | Chr01,  5,282,918 |
| *Xtr146* | CA635158 | 4.0e-48/84.70/353 | Chr05,  13,772,422 |
| *Xtr150* | TA8222_4565 | 0/82.62/1398 | Chr05,  18,102,163 |
| *Xtr248* | TA346_4565 | 1.0e-220/90.95/807 | Chr05,  1,379,517 |
| *Xtr232* | TA21859_4565 | 2.1e-130/90.42/167 | Chr02,  25,443,453 |
| *Xtr310* | TA22013_4565 | 3.2e-249/86.95/429 | Chr02,  1,276,832 |
| *Xtr329* | TA61249_4565 | 1.2e-110/83.96/293 | Chr01,  50,084,837 |
| *Xtr372* | TA82503_4565 | 2.9e-75/84.27/248 | Chr01,  26,858,852 |
| *Xtr383* | TA76259_4565 | 5.8e-90/87.67/292 | Chr01,  25,175,591 |
| *Xtr400* | TA69542_4565 | 3.9e-210/77.18/973 | Chr01,  58,787,698 |
| *Xtr451* | TA81179_4565 | 1.3e-123/77.73 /229 | Chr03,  21,487,679 |
| *Xtr471* | BG910093 | 3.1e-69/77.59/232 | Chr03,  12,739,569 |
| *Xtr537* | TA71452_4565 | 5.7e-08/62.56/422 | Chr03,  39,444,372 |
| *Xtr590* | TA72902_4565 | 4.5e-54/83.86/223 | Chr03,  35,864,339 |
| *Xtr615* | CJ552319 | 4.5e-75/81.47/313 | Chr01,  26,588,810 |
| *Xtr641* | TA109074_4565 | 6.2e-63/72.07/222 | Chr04,  15,477,515 |
| *Xtr764* | TA95943_4565 | 3.5e-92/72.61/449 | Chr04,  38,734,636 |
