# Supplementary material for: Syntenic Relationships between the U and M Genomes of Aegilops, Wheat and the Model Species Brachypodium and Rice as Revealed by COS Markers
Source: PLoS One. 2013 Aug 5;8(8):e70844. doi: 10.1371/journal.pone.0070844 (PMC3733919; doi:10.1371/journal.pone.0070844)
Supplement: Table S5 — PCR products of the COS markers in the genotypes of wheat and Aegilops species. (DOC) [file pone.0070844.s006.doc]

**Table S5**. COS markers used in the present study and the size of the PCR products amplified using DNA template from wheat (Chinese Spring, Mv9kr1, Mv25), *Aegilops umbellulata* (AE740/03, JIC2010001), *Ae. comosa* (MvGB1039, JIC2110001), *Ae. biuncialis* (MvGB642, MvGB382) and *Ae. geniculata* (TA2899, AE1311/00). The PCR products are given in bp.

| Marker | *T. aestivum* | | | *Ae. umbellulata* | | *Ae. comosa* | | *Ae. biuncialis* | | *Ae. geniculata* | |
| --- | --- | --- | --- | --- | --- | --- | --- | --- | --- | --- | --- |
|  | CS | Mv9kr1 | Mv25 | AE740/03 | 2010001 | MvGB1039 | 2110001 | MvGB642 | MvGB382 | 2899 | 1311/00 |
| *X1B* | 88, 230 | 88, 230 | 226, 230 | 224 | n.i. | 226 | n.i. | 226 | 226 | 226 | 226 |
| *X1D* | 181, 183 | 181, 183 | 181, 183 | 183 | n.i. | 183 | n.i. | 183 | 183 | 183 | 183 |
| *X1F* | 204, 208 | 204, 208 | 204, 208 | 204 | n.i. | 204 | n.i. | 204 | 204 | 204 | 204 |
| *X1J* | 215, 230 | 215, 230 | 215, 230 | 215 | n.i. | 228 | n.i. | 215, 228 | 215, 228 | 207, 215, 228 | 207, 215, 228 |
| *X1N* | 173 | 173 | 173 | 173 | n.i. | 174 | n.i. | 173 | 173, 174 | 173 | 173, 174 |
| *X1S* | 180, 190, 199 | 180, 190, 199 | 180, 190, 199 | 199 | n.i. | 190 | n.i. | 190, 199 | 190, 199 | 190, 199 | 190, 199 |
| *X2B* | 160, 167 | 160, 167 | 160, 167 | 162 | n.i. | 163 | n.i. | 162, 163 | 162, 163 | 162, 163 | 162, 163 |
| *X2C* | 160 | 160 | 160 | 159 | n.i. | 163 | n.i. | 159, 163 | 163 | 159 | 159 |
| *X2G* | 442, 445 | 445 | 445 | 445 | n.i. | 444 | n.i. | 427, 445 | 444, 445 | 445 | 444, 445 |
| *X2I* | 222 | 222 | 222 | 226 | n.i. | 230 | n.i. | 230 | 230 | 222, 230 | 228, 230 |
| *X2K* | 160, 171 | 160, 171 | 160, 171 | 160 | n.i. | 172 | n.i. | 160, 171 | 160, 172 | 160, 172 | 169, 172 |
| *X2N* | 551, 562, 582 | 551 | 551, 562, 582 | 558 | n.i. | 570 | n.i. | 547 | 558 | 549, 558 | 558 |
| *X2P* | 248, 276, 290 | 248, 276, 29 | 248, 276, 29 | 292 | n.i. | - | n.i. | 249, 273 | 249, 273 | 249, 292 | 249, 292 |
| *X2R* | 265, 342 | 265, 342 | 265, 342 | 265 | n.i. | 267 | n.i. | 265, 267 | 265, 267 | 265, 267 | 265, 267 |
| *X2U* | 353, 354 | 353, 354 | 353, 354 | 351 | n.i. | 353 | n.i. | 351, 353 | 351, 353 | 351 | 351, 353 |
| *X3B* | 198, 200, 231 | 198, 200, 231 | 198, 200, 231 | 234 | n.i. | 198 | n.i. | 198, 234 | 198, 234 | 232, 234 | 198, 234 |
| *X3F* | 197 | 197 | 197 | 197 | n.i. | 197 | n.i. | 197 | 197 | 197 | 197 |
| *X3H* | 194, 210 | 194, 210 | 194, 210 | 194 | n.i. | 210 | n.i. | 194, 210 | 194, 210 | 194, 228 | 194, 210 |
| *X3J* | 215 | 215 | 215 | 205 | n.i. | 215 | n.i. | 205, 215 | 205, 215 | 205, 215 | 205, 215 |
| *X3L* | 334, 354 | 334, 354 | 334, 354 | 353 | n.i. | 353 | n.i. | 353 | 353 | 353 | 353 |
| *X3N* | 302 | 302 | 302 | 302 | n.i. | 284 | n.i. | 302 | 284, 302 | 302 | 302 |
| *X3P* | 197, 204 | 197, 204 | 197, 204 | 198 | n.i. | 201 | n.i. | 198, 201 | 198, 201 | 198, 208 | 204 |
| *X3R* | 182 | 182 | 182 | 182 | n.i. | 182 | n.i. | 182 | 182 | 182 | 182 |
| *X3T* | 184 | 184 | 184 | 184 | n.i. | 184 | n.i. | 184 | 184 | 184 | 184 |
| *X4A* | 302 | 302 | 302 | 302 | n.i. | 302 | n.i. | 302 | 302 | 302 | 302 |
| *X4C* | 379, 381, 389, 397 | 379, 381, 389, 397 | 379, 381, 389, 397 | 385 | n.i. | 391, 399 | n.i. | 385, 395 | 370, 385, 393 | 378, 385, 395 | 385, 395 |
| *X4E* | 252, 263 | 252, 263 | 252, 263 | 263 | n.i. | 267 | n.i. | 261 | 267 | 248, 263 | 263 |
| *X4G* | 180, 182, 222, 227, 231 | 180, 182, 222, 227, 231 | 180, 182, 222, 227, 231 | 239 | n.i. | 235 | n.i. | 226, 228 | 226, 228 | 235, 239 | 226, 235 |
| *X4I* | 248 | 248 | 248 | 248 | n.i. | 249 | n.i. | 248, 249 | 248, 249 | 248, 249 | 248, 249 |
| *X4K* | 201, 202 | 201, 202 | 201, 202 | 201, 202 | n.i. | 201, 202 | n.i. | 201, 202 | 201, 202 | 201, 202 | 201, 202 |
| *X4M* | 286 | 286 | 286 | 286 | n.i. | 286 | n.i. | 286 | 286 | 286 | 286 |
| *X4O* | 213, 214 | 213, 214 | 213, 214 | 214 | n.i. | 212 | n.i. | 214, 217 | 212, 214 | 212, 214 | 212, 214 |
| *X4Q* | 213 | 213 | 213 | 212 | n.i. | 212 | n.i. | 212 | 212 | 212 | 212 |
| *X4S* | 362, 367, 372 | 362, 367, 372 | 362, 367, 372 | 372 | n.i. | 358 | n.i. | 370, 372 | 358, 372 | 370, 372 | 358, 372 |
| *X4U* | 167, 179, 183 | 167, 179, 183 | 167, 179, 183 | 180 | n.i. | 179 | n.i. | 179 | 167, 181 | 168, 180 | 179, 180 |
| *X5A* | 241, 243, 259 | 241, 243, 259 | 241, 243, 259 | 242, 259 | n.i. | 257, 259 | n.i. | 242, 245, 257, 259 | 242, 259 | 242, 245 | 242, 245, 259 |
| *X5C* | 386, 390, 392 | 386, 390 | 386, 390, 392 | 388 | n.i. | 392 | n.i. | 386, 388 | 386 | 386, 392 | 388 |
| *X5E* | 211, 215 | 211, 215 | 211, 215 | 211 | n.i. | 215 | n.i. | 211, 215 | 211, 215 | 211, 236 | 211, 215, 236 |
| *X5I* | 274, 280 | 274, 280 | 274, 280 | 270 | n.i. | 274 | n.i. | 270, 274 | 270, 274 | 270, 272 | 270, 274 |
| *X5G* | 159 | 159 | 159 | 159 | n.i. | 159 | n.i. | 159 | 159 | 156, 159 | 159 |
| *X5K* | 204 | 204 | 204 | 204 | n.i. | 204 | n.i. | 203, 204 | 203, 204 | 203, 204 | 203, 204 |
| *X5M* | 205, 217 | 205, 209, 217 | 205, 209, 217 | - | n.i. | - | n.i. | 195, 205, 209 | - | 199, 205, 209 | - |
| *X5O* | - | - | - | - | n.i. | - | n.i. | - | - | - | - |
| *X5Q* | 309, 359 | 309, 359 | 309, 359 | 311 | n.i. | 311 | n.i. | 311 | 311 | 311 | 311 |
| *X5S* | 446, 450 | 446, 450 | 446, 450 | 451 | n.i. | - | n.i. | 450 | 450 | 443, 451 | 443, 486 |
| *X5V* | 397, 419 | 397, 419 | 397, 419 | 397, 418 | n.i. | 397 | n.i. | 397, 418 | 397, 418 | 397, 418 | 397, 418 |
| *X6P* | 141, 320 | 141, 320 | 141, 320 | 141, 320 | n.i. | 141, 320 | n.i. | 141, 320 | 141, 320 | 141, 320 | 141, 320 |
| *X6R* | 288, 327 | 288, 327 | 288, 327 | - | n.i. | 288 | n.i. | 288 | 288 | 327 | 288 |
| *X6A* | 253, 267, 271 | 253, 267, 271 | 253, 267, 271 | 250, 277 | n.i. | 263 | n.i. | 250, 262, 269, 277 | 250, 277, 281, 290 | 250, 267, 273, 277 | 250, 269, 277, |
| *X6C* | - | - | - | - | n.i. | - | n.i. | no amp | - | - | - |
| *X6E* | 206 | 206 | 206 | 207 | n.i. | 205 | n.i. | 205, 207 | 205, 207 | 205, 207 | 205, 207 |
| *X6N* | 450, 452 | 450, 452, 455 | 450, 452, 455 | 216 | n.i. | 411, 440 | n.i. | 216 | 216, 440 | 216, 491 | 216, 440 |
| *X6O* | 264, 300 | 264, 300 | 264, 300 | 260 | n.i. | 266 | n.i. | 260, 264 | 260, 264 | 260 | 260, 264 |
| *X6J* | 239, 243 | 239, 243 | 239, 243 | 236 | n.i. | 236 | n.i. | 236 | 236 | 236, 238 | 236 |
| *X6L* | 308, 309, 310 | 308, 309, 310 | 308, 309, 310 | 307 | n.i. | 309 | n.i. | 307, 309 | 307, 309 | 308, 309 | 307, 309 |
| *X7A* | 238 | 238 | 238 | 238 | n.i. | 238 | n.i. | 238 | 238 | 238 | 238 |
| *X7C* | 316, 322 | 316, 322 | 316, 322 | 327 |  | 328 |  | 319, 328 | 327, 328 | 322, 327 | 327, 328 |
| *X7E* | 225, 233, 239 | 225, 233, 239 | 225, 233, 239 | 239 | 239 | 233 | 233 | 239 | 233, 239 | 239 | 233, 239 |
| *X7G* | - | - | - | - | - | - | - | - | - | - | - |
| *X7I* | 262, 263, 272 | 262, 263, 272 | 262, 263, 272 | 248, 263 | 248, 263 | 249, 312 | 249, 312 | 248, 249, 263, 312 | 248, 249, 263, 312 | 245, 249, 262, 312 | 248, 249, 263 , 312 |
| *X7L* | 437, 439 | 437, 439 | 437, 439 | 439 | 439 | 438 | 438 | 438, 439 | 438, 439 | 437, 439 | 438, 439 |
| *X7T* | 282, 286, 292, 299, 305, 307 | 282, 286, 292, 299, 305, 307 | 282, 286, 292, 299, 305, 307 | 283, 296 | 283, 296 | 296, 299 | 299, 312 | 283, 296, 314 | 283, 296, 305 | 283, 296, 301, 304, 314 | 283, 299, 314 |
| *Xtr4* | 236, 284 | 236, 268, 284 | 236, 268, 284 | 250, 266 | 266 | 280 | 247 | 272 | 266, 280 | 267, 271, 273, 281 | 271, 281 |
| *Xtr60* | 241, 248 | 241, 248 | 241, 248 | 242 | 242 | 240, 241 | 240, 241 | 241, 242 | 241, 242 | 229, 241, 242 | 241, 242 |
| *Xtr61* | 346, 366 | 365, 366, 451 | 346, 366, 451 | 367 | 347,367 | 461 | 461 | 338, 365, 367 | 367, 461 | 364, 367 | 367 |
| *Xtr62* | 175 | 175 | 175 | 180 | 180 | 178 | 178 | 178, 180 | 178, 180 | 178, 180 | 178, 180 |
| *Xtr63* | 434, 450 | 434, 450 | 434, 450 | 545 | 545 | 444 | 444 | 444, 545 | 444, 545 | 435, 545 | 444, 545 |
| *Xtr64* | 282, 284, 287 | 282, 284 |  | 282 | 282 | 282 | 282 | 282 | 282 | 282 | 282 |
| *Xtr66* | 371, 373, 377 | 371, 373, 377 | 371, 373, 377 | 376 | 376 | 376 | 376 | 376 | 376 | 376 | 376 |
| *Xtr67* | 347, 348 | 347, 348 | 347, 348 | 349 | 349 | 343 | 343, 351 | 349, 351, 352 | 349, 351 | 343, 349 | 350, 351 |
| *Xtr68* | 370, 376 | 370, 376 | 370, 376 | 375 | 375 | 367 | 367 | 370, 375 | 367, 370, 375 | 370, 375 | 367, 375 |
| *Xtr70* | 257 | 257 | 257 | 257 | 261 | 260 | 260 | 257 | 260, 261 | 257 | 257, 260 |
| *Xtr71* | 556 | 556 | 556 | 556 | 556 | 556 | 556 | 556 | 556 | 556 | 556 |
| *Xtr72* | 152, 157, 190, 227, 256 | 152, 157, 190, 227, 258 | 152, 157, 190, 227, 258 | 179 | 179, 237 | 168, 259 | 168, 259 | 168, 179 | 179, 259 | 179, 237, 261 | 168, 179 |
| *Xtr73* | 509, 512 | 509, 512 | 476, 500, 509, 512, 521 | 485 | 485, 509 | 473 | 473 | 485 | 485 | - | 481 |
| *Xtr76* | 152, 157, 190, 227, 257 | 152, 157, 190, 227, 258 | 152, 157, 190, 227, 258 | 179, 237 | 179, 237 | 168, 259 | 168, 259 | 168, 179, 237, 259 | 179, 237, 259 | 179, 237, 261 | 168, 179, 237, 259 |
| *Xtr77* | 285, 288, 293 | 285, 288, 293 | 285, 288, 293 | 290, 363 | 290, 363 | 293 | 286 | 290, 364 | 293, 363 | 294, 364 | 288, 293 |
| *Xtr80* | 415, 421, 459 | 415, 421, 459 | 415, 421, 459 | 429 | 429 | 487 | 487 | 429, 487 | 429, 487 | 429, 446 | 429, 487 |
| *Xtr81* | 360 | 360 | 360 | 360 | 360 | 371 | 371 | 360 | 360, 371 | 360 | 360, 371 |
| *Xtr82* | 329 | 329 | 329 | 329 | 329 | 325 | 329 | 325, 329 | 325, 329 | 329 | 325, 329 |
| *Xtr83* | 353, 358 | 353, 358 | 353, 358 | 360 | 360 | 356 | 356 | 356, 360 | 356, 360 | 353, 360 | 356, 360 |
| *Xtr85* | 219, 226 | 215, 219 | 215, 219 | 215 | 215 | 226 | 226 | 215, 226 | 215, 226 | 215, 219 | 215, 226 |
| *Xtr87* | - | - | - | - | - | 431 | 431 | - | 431 | - | 431 |
| *Xtr88* | 402 | 402 | 402 | - | - | - | 407 | 407 | 407 | 407 | 407 |
| *Xtr90* | 264, 282, 292 | 264, 282, 292 | 264, 282, 292 | 290 | 290 | 290 | 291, 305 | 291 | 291 | 274, 290, 291 | 290, 291 |
| *Xtr91* | 329, 334, 339 | 329, 334, 339 | 329, 334, 339 | 287 | 287 | 335 | 315 | 287, 335 | 287, 335 | 287, 335 | 287, 335 |
| *Xtr92* | 203, 228, 238 | 203, 238 | 203, 228, 238 | 231 | 231 | 229 | 228 | 229, 231 | 229, 231 | 229, 231 | 229, 231 |
| *Xtr93* | 481, 487 | 481, 487 | 481, 487 | 487 | 487 | 477 | 477 | 477, 487 | 488 | 475, 488 | 475, 488 |
| *Xtr94* | 223, 224, 255 | 223, 224, 255 | 223, 224, 255 | 255 | 255 | 255 | 255 | 255 | 255 | 255 | 255 |
| *Xtr96* | 248, 258 | 248, 258 | 248, 258 | 239 | 239 | 258 | 258 | 258 | - | - | - |
| *Xtr97* | 240 | 240 | 240 | 240 | 240 | 240 | 240 | 240 | 240 | 240 | 240 |
| *Xtr99* | 374, 378 | 374, 378 | 374, 378 | 378 | 378 | 378 | 378 | 378 | 378 | 378 | 378 |
| *Xtr100* | 460 | 460 | 460 | 459 | 460 | 460 | 460 | 459, 461 | 459, 462 | 459, 461 | 459, 462 |
| *Xtr101* | 177, 178 | 177, 178 | 177, 178 | 178 | 178 | 178 | 178 | 178 | 178 | 178 | 178 |
| *Xtr102* | 305, 316 | 305, 316 | 305, 316 | 318 | 318 | 305 | 305 | 305, 318 | 305, 318 | 316, 318 | 305, 318 |
| *Xtr103* | 267, 268 | 267, 268 | 267, 268 | 270 | 261, 270 | 261 | 261 | 261, 270 | 261, 270 | 261, 270 | 261, 270 |
| *Xtr104* | 402, 424 | 402, 424 | 402, 424 | 423 | 423 | 406 | 406 | 406, 423 | 406, 423 | 366, 406, 423 | 406, 423 |
| *Xtr105* | 262, 263, 275 | 262, 263, 275 | - | 262 | 262 | 279 | 279 | - | 262, 279 | 262 | 262, 279 |
| *Xtr106* | 239 | 240 | 239 | 241 | 239 | 239 | 239 | 240, 241 | 240, 262 | 240 | 239 |
| *Xtr107* | 460 | 460 | - | 471 | 471 | 468 | 466 | 471 | 471 | 471 | 465, 471 |
| *Xtr108* | - | - | - | - | - | - | - | - | - | - | - |
| *Xtr110* | 360, 362 | 360, 362 | 360, 362 | 362 | 362 | 358 | 362 | 358, 362 | 358, 362 | 362 | 362 |
| *Xtr112* | 384, 387, 395 | 387, 395 | 387, 395 | 384 | 384 | 390 | 390 | 384, 387 | 384, 387 | 383, 387 | 384, 387 |
| *Xtr126* | 402 | 402 | 402 | 402 | 402 | 499 | 499 | 402 | 402 | 402 | 402 |
| *Xtr128* | 207, 255 | 207, 255 | 207, 255 | 214 | 214 | 197 | 212 | 214, 216 | 212, 214 | 210, 214 | 213 |
| *Xtr129* | 236, 238, 241 | 236, 238, 241 | 236, 238, 241 | 242 | 242 | 240 | 240 | 240, 242 | 240, 242 | 242, 300 | 240, 300 |
| *Xtr131* | 346, 347, 395 | 346, 347, 395 | 346, 347, 395 | 470 | 470 | 356 | 356 | 356, 464 | 356, 470 | 396, 470 | 354, 470 |
| *Xtr134* | 255 | 255 | 255 | 255 | 255 | 250 | 250 | 250, 255 | 250, 255 | 255 | 250, 255 |
| *Xtr135* | 254, 260, 262 | 254, 260, 262 | 254, 260, 262 | 262 | 262 | 260 | 260 | 260, 262 | 258, 260 | 260, 262 | 260, 262 |
| *Xtr143* | 337, 367 | 337, 367 | 337, 367 | 367 | 367 | 367 | 367 | 367 | 367 | 367 | 367 |
| *Xtr146* | 231, 388 | 231, 388 | 231, 388 | 303 | 303 | 381 | 381 | 303, 381 | 303, 381 | 303, 381 | 303, 381 |
| *Xtr150* | 199, 205 | 199, 201, 205 | 199, 201, 205 | 205 | 205 | 201 | 201 | 201, 205 | 205 | 201, 205 | 205 |
| *Xtr154* | 229, 231 | 229, 231 | 229, 231 | 229 | 229 | 229 | 229 | 229 | 229 | 230 | 229 |
| *Xtr170* | - | - | - | - | - | - | - | - | - | - | - |
| *Xtr232* | 380, 382 | 380, 382 | 380, 382 | 382 | 382 | 382 | 382 | 382 | 382 | 382 | 382 |
| *Xtr248* | 216, 382 | 216, 382 | 216, 382 | 208 | 137 | 201, 391 | 391 | 208 | 391 | 208, 216 | 201 |
| *Xtr310* | 258 | 258 | 258 | 258 | 258 | 257 | 257 | 258 | 258 | 258 | 258 |
| *Xtr329* | 258 | 258 | 258 | 257 | 257 | 258 | 258 | 257, 259 | 257, 258 | 258 | 257, 258 |
| *Xtr330* | 258 | 258 | 258 | 258 | 258 | 258 | 258 | 258 | 258 | 258 | 258 |
| *Xtr366* | 216, 252, 255 | 216, 255 | 216, 252, 255 | 216, 255 | 216, 255 | 216, 255 | 216, 255 | 216, 255 | 216, 255 | 216, 255, 256 | 216, 255 |
| *Xtr372* | 215, 222 | 215, 222 | 215, 222 | 216 | 216 | 216 | 216 | 216 | 216 | 216 | 216 |
| *Xtr383* | 164, 169, 183 | 164, 169, 183 | 164, 169, 183 | 165 | 165 | 183 | 183 | 165, 183 | 165, 189 | 165, 186 | 165, 183 |
| *Xtr393* | - | - | - | - | - | - | - | - | - | - | - |
| *Xtr400* | 102, 121, 147 | 102, 121, 147 | 102, 121, 147 | 127, 147 | 147 | 125, 147 | 125, 147 | 125, 127, 147 | 147 | 102, 127, 147 | 147 |
| *Xtr413* | 277 | 277 | 277 | 277 | - | 277 | - | 277 | 277 | 277 | 277 |
| *Xtr437* | 264 | 264 | 264 | 265 | 265 | 265 | 265 | 265 | 265 | 265 | 265 |
| *Xtr451* | 252, 265 | 252 | 252, 265 | 262 | 192, 262 | 264 | 264 | 262, 264 | 262, 264 | 258, 262, 264 | 262, 264 |
| *Xtr462* | 161 | 161 | 161 | 161 | 161 | 161 | 161 | 161 | 161 | 161 | 161 |
| *Xtr471* | 177, 254, 273, 276 | 177, 254, 273, 276 | 177, 254, 273, 276 | 254, 276 | 254, 276 | 209, 270 | 209 | 209, 254, 263, 270, 276 | 209, 254, 270, 276 | 170, 209, 254, 276 | 209, 254, 270, 276 |
| *Xtr488* | 154 | 154 | 154 | 154 | 154 | 154 | 154 | 154 | 154 | 154 | 154 |
| *Xtr537* | 255 | 255 | 255 | 254 | 254 | 260 | 260 | 254 | 254, 260 | 254 | 254 |
| *Xtr570* | 261 | 261 | 261 | 261 | 261 | 261 | 261 | 261 | 261 | 261 | 261 |
| *Xtr590* | 201 | 201 | 201 | 201 | 201 | 200 | 200 | 200, 201 | 200, 201 | 200, 201 | 200, 201 |
| *Xtr615* | 205 | 205 | 205 | 204 | 205 | 204 | 204 | 204, 205 | 204 | 204, 205, 206 | 204, 205 |
| *Xtr641* | 248, 332 | 248, 332 | 248, 332 | 248 | 248 | 247 | 247 | 247, 248 | 247, 248 | 248 | 247, 248 |
| *Xtr654* | 255, 288 | 255, 288 | 255, 288 | 255 | 255 | 255 | 255 | 255, 257 | 255 | 255 | 255 |
| *Xtr725* | - | - | - | - | - | - | - | - | - | - | - |
| *Xtr731* | 378, 380 | 378, 380 | 378, 380 | 380 | 380 | 380 | 380 | 380, 385 | 380, 385 | - | 380, 385 |
| *Xtr757* | 187, 190, 193 | 187, 190, 193 | 187, 190, 193 | 187 | 187, 190 | 190 | 190 | 187, 190 | 187, 190 | 187 | 187, 190 |
| *Xtr764* | 197, 207 | 197, 207, 222 | 197, 207, 222 | 207 | 207 | 214 | 214 | 207, 214 | 207, 214 | 207, 214 | 207, 214 |

n.i.: Not investigated

-: No PCR product was detected
