# Supplementary material for: Syntenic Relationships between the U and M Genomes of Aegilops, Wheat and the Model Species Brachypodium and Rice as Revealed by COS Markers
Source: PLoS One. 2013 Aug 5;8(8):e70844. doi: 10.1371/journal.pone.0070844 (PMC3733919; doi:10.1371/journal.pone.0070844)
Supplement: Table S6 — Assignment of COS markers to the chromosomes or to the peaks on flow karyotypes in Aegilops umbellulata, Ae. comosa, Ae. biuncialis and Ae. geniculata . (DOC) [file pone.0070844.s007.doc]

**Table S6.** PCR amplicons of COS markers (in bp) assigned to the chromosomes or to the peaks on flow karyotypes (I-IV) in different genotypes of *Aegilops umbellulata*, *Ae. comosa*, *Ae. biuncialis* and *Ae. geniculata*.

| *Marker* | *Ae. umbellulata* | | *Ae. comosa* | | *Ae. biuncialis* | | *Ae. geniculata* | |
| --- | --- | --- | --- | --- | --- | --- | --- | --- |
|  | AE740/03 | JIC2010001 | MvGB1039 | JIC2110001 | MvGB642 | MvGB382 | TA2899 | AE1311/00 |
| *X1B* | 224 (I=1U) | n.i. | 226 (I) | n.i. | 226 (1U) | 226 (I=1U, III) | 226 (1U, 1M) | 226 (I) |
| *X1D* | 183 (I=1U) | n.i. | 183 (I) | n.i. | 183^np^ | 183 (I=1U) | 183^np^ | 183 (I) |
| *X1F* | 204 (I=1U) | n.i. | 204 (I) | n.i. | 204 ^np^ | 204 (I=1U) | 204 ^np^ | 204 (I) |
| *X1J* | 215 (I=1U) | n.i. | 228 (III) | n.i. | 215(-),  228(-) | 215 (I=1U),  228 (II) | 207 (1M),  215(-),  228 (2M) | 207 (IV),  215 (I),  228 (III) |
| *X1N* | 173 (I=1U) | n.i. | 174 (I) | n.i. | 173^np^ | 173 (I=1U),  174 (III) | 173^np^ | 173(I),  174(II) |
| *X1S* | 199 (IV) | n.i. | 190 (IV) | n.i. | 190 ^np^,  199 ^np^ | 190 (II),  199 (IV) | 190 ^np^,  199 ^np^ | 190 (IV),  199 (III) |
| *X2B* | 162 (I=1U) | n.i. | 163 (I) | n.i. | 162 (1U),  163(-) | 162 (I=1U),  163 (III) | 162 (1U),  163 (1M) | 162 (I),  163 (IV) |
| *X2C* | 159 (II=6U) | n.i. | 163 (I) | n.i. | 159 (6U),  163(-) | 163 (II) | 159 | 159 (II) |
| *X2G* | 445 (IV) | n.i. | 444 (IV) | n.i. | 427(-),  445 ^np^ | 444 (III),  445 (III) | 445 ^np^ | 444 (III),  445 (III) |
| *X2I* | 226 (II=6U) | n.i. | 230 (III) | n.i. | 230 (6U) | 230 (II) | 222 ^np^,  230 (2M) | 228 (II),  230 (III) |
| *X2K* | 160 (IV) | n.i. | 172 (III) | n.i. | 160 ^np^,  171(-) | 160 (III),  172 (II) | 160 ^np^,  172 (2M) | 169 (III),  172 (III) |
| *X2N* | 558 (IV) | n.i. | 570 (IV) | n.i. | 547(-) | 558 (III) | 549(-),  558 (2U) | 558 (III) |
| *X2P* | 292 (IV) | n.i. | - | n.i. | 249(-),  273(-) | 249 (II),  273 (III) | 249 (2M),  292 (2U) | 249 (III),  292 (III) |
| *X2R* | 265 (IV) | n.i. | 267 (-) | n.i. | 265 ^np^,  267(-) | 265 (III),  267 (II) | 265 ^np^,  267 (2M) | 265 (III),  267 (III) |
| *X2U* | 351 (IV) | n.i. | 353 (I) | n.i. | 351 (6U),  353(-) | 351 (II),  353 (III) | 351 (2U, 2M) | 351 (III) |
| *X3B* | 234 (IV) | n.i. | 198 (IV) | n.i. | 198 ^np^,  234(-) | 198 (IV),  234 (III) | 234 (7U) | 198 (IV),  234 (III) |
| *X3F* | 197 (III=3U) | n.i. | 197 (IV) | n.i. | 197 ^np^ | 197 (II, III) | 197 ^np^ | 197 (II, IV) |
| *X3H* | 194 (III=3U) | n.i. | 210 (IV) | n.i. | 194 ^np^,  210 ^np^ | 194 (II),  210 (III) | 194 ^np^ | 194 (II),  210 (IV) |
| *X3J* | 205 (III=3U) | n.i. | 215 (IV) | n.i. | 205 (3U),  215 ^np^ | 205 (II),  215 (III) | 205 (3U),  215 ^np^ | 205 (II),  215 (IV) |
| *X3L* | 353 (III=3U) | n.i. | 353 (IV) | n.i. | 353(-) | 353 (II, III) | 353(-) | 353 (II, IV) |
| *X3N* | 302 (III=3U) | n.i. | 284 (IV) | n.i. | 302 ^np^ | 284 (III),  302 (II) | 302 ^np^ | 302 (II) |
| *X3P* | 198 (III=3U) | n.i. | 201 (IV) | n.i. | 198 (3U),  201(-) | 198 (II),  201 (III) | 198(-),  208(-) | 204 (II) |
| *X3R* | 182 (III=3U) | n.i. | 182 (IV) | n.i. | 182 ^np^ | 182 (III) | 182 ^np^ | 182 (II, IV) |
| *X3T* | 184 (III=3U) | n.i. | 184 (IV) | n.i. | 184 ^np^ | 184 (II, III) | 184 ^np^ | 184 (II, IV) |
| *X4A* | 302 (IV) | n.i. | 302 (IV) | n.i. | 302 ^np^ | 302 (III) | 302 ^np^ | 302 (III) |
| *X4C* | 385 (II=6U) | n.i. | 391 (I),  399 (I) | n.i. | 385 (6U),  395(-) | 385 (II),  393 (II) | 385(-),  395(-) | 385 (II),  395 (I) |
| *X4E* | 263 (-) | n.i. | 267 (I) | n.i. | 261 (2M, 3M) | 267 (II) | 248(-),  263 ^np^ | 263 (-) |
| *X4G* | 239 (II=6U) | n.i. | 235 (I) | n.i. | 226 (6U),  228 (2M, 3M) | 226 (II),  228 (II) | 235(-),  239(-) | 226 (II),  235 (-) |
| *X4I* | 248 (II=6U) | n.i. | 249 (I) | n.i. | 248(-),  249 (2M, 3M) | 248 (I=1U),  249 (II) | 248 ^np^,  249(-) | 248 (II),  249 (I) |
| *X4K* | 201 (II=6U), | n.i. | 201 (I), | n.i. | 201 ^np^, | 201 (II), | 201 ^np^, | 201 (II), |
| *X4M* | 286 (IV) | n.i. | 286 (I) | n.i. | 286 ^np^ | 286 (II) | 286 ^np^ | 286 (III) |
| *X4O* | 214 (IV) | n.i. | 212 (I) | n.i. | 214(-),  217 (2M, 3M) | 212 (II),  214 (III) | 212(-),  214(-) | 212 (IV),  214 (III) |
| *X4Q* | 212 (IV) | n.i. | 212 (I) | n.i. | 212 (2M, 3M) | 212 (II, III) | 212(-) | 212 (III) |
| *X4S* | 372 (IV) | n.i. | 358 (I) | n.i. | 370 (2M, 3M), 372 ^np^ | 358 (II),  372 (III) | 370(-),  372(-) | 358 (IV),  372 (III) |
| *X4U* | 180 (IV) | n.i. | 179 (I) | n.i. | 179(-) | 167 (II),  181 (III) | 168(-),  180(-) | 179 (IV),  180 (III) |
| *X5A* | 242 (IV),  259 (II=6U) | n.i. | 257 (II),  259 (IV) | n.i. | 242(-),  245(-),  257(-),  259(-) | 242 (III),  259 (III) | 242 (5U),  245 (5M) | 242 (III),  245 (III),  259 (IV) |
| *X5C* | 388 (IV) | n.i. | 392 (II) | n.i. | 386 ^np^,  388(-) | 386 (III) | 386 ^np^,  392 ^np^ | 388 (III) |
| *X5E* | 211 (IV) | n.i. | 215 (II) | n.i. | 211 ^np^,  215 ^np^ | 211 (III),  215 (II) | 211 ^np^, 236 | 211 (III),  215 (III),  236 (I) |
| *X5I* | 270 (IV) | n.i. | 274 (IV) | n.i. | 270(-),  274 ^np^ | 270 (III),  274 (II) | 270 (5U),  272(-) | 270 (III),  274 (III) |
| *X5G* | 159 (IV) | n.i. | 159 (IV) | n.i. | 159 ^np^ | 159 (III) | 159 ^np^ | 159 (III) |
| *X5K* | 204 (IV) | n.i. | 204 (III) | n.i. | 203(-) | 203 (III) | 203 (5M) | 203 (III) |
| *X5M* | × | n.i. | × | n.i. | 195(-),  205 ^np^,  209 ^np^ | × | 199 (5U)  205 ^np^,  209 (4U, 7M) | × |
| *X5O* | × | n.i. | × | n.i. | × | × | × | × |
| *X5Q* | 311 (IV) | n.i. | 311 (III) | n.i. | 311(-) | 311 (III) | 311 (5U, 5M) | 311 (III) |
| *X5S* | 451 (IV) | n.i. | × | n.i. | 450(-) | 450 (III) | 443 (5U),  451(-) | 443 (III),  486(-) |
| *X5V* | 397 (IV),  418 (IV) | n.i. | 397 (IV) | n.i. | 397 ^np^,  418(-) | 397 (III),  418 (III) | 397 ^np^,  418(-) | 397 (III),  418 (II) |
| *X6P* | 141 (-),  320 (-) | n.i. | 141 (IV),  320 (-) | n.i. | 141 ^np^,  320 ^np^ | 141 (III),  320 (-) | 141 ^np^,  320 ^np^ | 141 (-),  320 (-) |
| *X6R* | bad PCR | n.i. | 288 (II) | n.i. | 288 ^np^ | 288 (II) | 327 ^np^ | 288 (I) |
| *X6A* | 250 (II=6U)  277 (IV) | n.i. | 263 (IV) | n.i. | 250 (6U),  262 (7M),  277(-) | 250 (II),  277 (III),  281 (IV),  290 (II) | 250 (7M),  267 ^np^,  273(-),  277 (7U) | 250 (IV),  269 (IV),  277 (III) |
| *X6C* | × |  | × |  | × | × | × | × |
| *X6E* | 207 (IV) | n.i. | 205 (II) | n.i. | 205(-),  207(-) | 205 (II),  207 (III) | 205 (6M),  207(-) | 205 (I),  207 (III) |
| *X6N* | 216 (I=1U,  III=3U) | n.i. | 411 (I),  440 (II) | n.i. | 216 (3U, 2M, 3M, 7M | 216 (I=1U),  440 (II) | 216 (1U, 2U, 7U, 1M, 2M),  491(-) | 216 (I=1U),  440 (I=6M) |
| *X6O* | 260 (IV) | n.i. | 266 (II) | n.i. | 260(-),  264 ^np^ | 260 (III),  264 (II) | 260(-) | 260 (III),  264 (I=6M) |
| *X6J* | 236 (IV) | n.i. | 236 (II) | n.i. | 236(-) | 236 (II, III) | 236 (4U, 6M), | 236 (I=6M, III) |
| *X6L* | 307 (IV) | n.i. | 309 (II) | n.i. | 307(-),  309 ^np^ | 307 (III),  309 (II) | 309 ^np^ | 307 (III),  309 (I=6M) |
| *X7A* | 238 (IV) | n.i. | 238 (IV) | n.i. | 238 ^np^ | 238 (III) | 238 ^np^ | 238 (III) |
| *X7C* | 327 (IV) |  | 328 (IV) |  | 319(-),  328 (7M) | 327 (III),  328 (IV) | 322 ^np^,  327 (7U) | 327 (III),  328 (IV) |
| *X7E* | 239 (III=3U) | 239 ^np^ | 233 (IV) | 233 ^np^ | 239 ^np^ | 233 (III),  239 (II) | 239 ^np^ | 233 (IV),  239 (II) |
| *X7I* | 248 (IV),  263 (IV) | 248 (7U),  263^np^ | 249 (IV),  312 (IV) | 249 (7M),  312 (7M) | 248(-),  249 (7M),  263 ^np^,  312 (7M) | 248 (III),  249 (IV),  263 (IV),  312 (IV) | 249 (7M),  262 ^np^,  312 (7M) | 249 (IV),  263 (III),  312 (IV) |
| *X7L* | 439 (IV) | 439^np^ | 438 (IV) | 438 (4M, 7M) | 438 (7M),  439 ^np^ | 438 (IV),  439 (III) | 438 (7U, 7M), 439 ^np^ | 438 (IV),  439 (III) |
| *X7T* | 283 (II=6U),  296 (IV) | 283(-),  296(-) | 296 (II),  299 (IV) | 299 ^np^,  312 (6M) | 283 (6U),  296(-),  314(-) | 283 (II),  296 (II),  305 (II) | 283(-),  296 (6M),  301(-),  304(-),  314 (3U, 4U, 2M, 7M) | 283 (II),  299 (IV),  314 (III) |
| *Xtr4* | 250 (IV),  266 (IV) | 266 (7U) | 280 (IV) | 247(-) | 272(-) | 266 (III),  280 (IV) | 267(-),  271 (7U),  273(-),  281 (7U) | 271 (III),  281 (III) |
| *Xtr60* | 242 (III=3U) | 242(-) | 241 (IV) | 241(-) | 241(-),  242 (3U) | 241 (II),  242 (II) | 241(-),  242(3U) | 241 (IV),  242 (II) |
| *Xtr61* | 367 (IV) | 347 (4U),  367 (4U, 7U) | 461 (IV) | - | 338(-),  365(-),  367 (3M) | 367 (III),  461 (II) | 364(-),  367 (4U, 7U) | 367 (III) |
| *Xtr62* | 180 (III=3U) | 180(-) | 178 (IV) | 178 (3M) | 178(-),  180 (3U) | 178 (II),  180 (II) | 178(-),  180 (3U) | 178 (IV),  180 (II) |
| *Xtr63* | 545 (III=3U) | 545(-) | 444 (IV) | 444 (3M) | 444(-),  545 (3U) | 444, (II)  545 (II) | 435(-),  545 (3U) | 444 (IV),  545 (II) |
| *Xtr64* | 282 (II=6U) | 282 (6U) | 282 (-) | 282 (-) | 282^np^ | 282 (bp) | 282 ^np^ | 282 (I, II) |
| *Xtr66* | 376 (III=3U) | 376 (-) | 376 (IV) | 376 (3M) | 376 (3U) | 376 (bp) | 376 (3U, 7M) | 376 (II) |
| *Xtr67* | 349 (bp) | 349 (-) | 343 (IV) | 343 (-),  351 (3M) | 349 (3U),  351 (-), | 349 (bp),  351 (bp) | 343 (-),  349 (-) | 350 (II),  351 (IV) |
| *Xtr68* | 375 (III=3U) | 375 (-) | 367 (IV) | 367 (-) | 370 (-),  375 (3U) | 367 (bp),  370 (bp),  375 (bp) | 370 (-),  375 (3U) | 367 (IV),  375 (II) |
| *Xtr70* | 257 (III=3U) | 261 (-) | 260 (IV) | 260 (-) | 257^np^ | 260 (bp),  261 (bp) | 257 ^np^ | 257 (II),  260 (IV) |
| *Xtr71* | 556 (III=3U) | 556 (-) | 556 (-) | 556 (-) | 556 ^np^ | 556 (bp) | 556 ^np^ | 556 (II, IV) |
| *Xtr72* | 179 (IV) | 179 (4U),  237 (-) | 168 (II) | 168 (-),  259 (3M) | 168 (2M, 3M), 179 (-) | 179 (III),  259 (III) | 179 (4U),  237 (-),  261 (-) | 168 (IV),  179 (II) |
| *Xtr73* | 485 (bp) | 485 (-), | 473 (IV) | 473 (3M) | 485 (-) | 485 (IV) | × | 481 (IV) |
| *Xtr76* | 179 (IV),  237 (III=3U) | 179 (4U),  237 (-) | 259 (IV) | 168 (-),  259 (3M) | 168 (2M, 3M), 179 (-),  237 (-),  259 (-) | 179 (III),  259 (III) | 179 (4U),  237 (-), | 168 (IV),  179 (II), |
| *Xtr77* | 290 (IV),  363 (II=6U) | 290 (-), | 293 (IV) | 286 (3M) | 290 (-),  364 (3U) | 293 (III),  363 (II) | 294 (-),  364 (-) | 288 (II),  293 (IV) |
| *Xtr80* | 429 (III=3U) | 429 (-) | 487 (IV) | 487 (3M) | 429 (3U),  487 (-) | 429 (II),  487 (III) | 429 (3U),  446 (-) | 429 (II),  487 (IV) |
| *Xtr81* | 360 (III=3U) | 360^np^ | 371 (IV) | 371 (-) | 360^np^ | 360 (II),  371 (III) | 360 ^np^ | 360 (III),  371 (IV) |
| *Xtr82* | 329 (III=3U) | 329 ^np^ | 325 (IV) | - | 325 (-),  329^np^ | 325 (III),  329 (II) | 329^np^ | 325 (IV),  329 (II) |
| *Xtr83* | 360 (III=3U) | 360 (-) | 356 (IV) | 356 (3M) | 356 (-),  360 (3U) | 356 (III), | 353^np^,  360 (3U) | 356 (IV),  360 (II) |
| *Xtr85* | 215 (IV) | 215 (7U) | 226 (IV) | 226 (-) | 215^np^,  226 (3M) | 215 (III),  226 (III) | 215 (7U),  219^np^ | 215 (III),  226 (IV) |
| *Xtr87* | × | × | 431 (I) | 431 (-) | × | 431 (II) | × | 431 (bp) |
| *Xtr88* | × | × | × | 407 (4M) | 407 (-) | 407 (II) | 407 (-) | 407 (III) |
| *Xtr90* | 290 (II=6U) | 290 (6U) | 290 (II) | 291 (-),  305 (6M) | 291 (6U) | 291 (II) | 290 (-),  291 (6M) | 290 (II),  291 (I) |
| *Xtr91* | 287 (II=6U) | 287 (6U) | 335 (II) | 315 (6M) | 287 (6U),  335 (-) | 287 (II),  335 (II) | 287 (-),  335 (6M) | 287 (II),  335 (I=6M) |
| *Xtr92* | 231 (IV) | 231 (4U) | 229 (-) | 228^np^ | 229 (-),  231 (-) | 229 (II),  231 (III) | 229 (-),  231 (4U) | 229 (bp),  231 (II) |
| *Xtr93* | 487 (I=1U?) | 487^np^ | 477 (II) | 477 (6M) | 477 (-),  487^np^ | 488 (II) | 475 (6M),  488^np^ | 475 (I=6M),  488 (II) |
| *Xtr94* | 255 (IV) | 255 ^np^ | 255 (II) | 255 ^np^ | 255^np^ | 255 (III) | 255^np^ | 255 (III) |
| *Xtr96* | 239 (bp) | 239 (-) | 258 (-) | 258 (7M) | 258^np^ | × | × | × |
| *Xtr97* | 240 (IV) | 240 ^np^ | 240 (II) | 240 ^np^ | 240 ^np^ | 240 (II) | 240 ^np^ | 240 (I) |
| *Xtr99* | 378 (IV) | 378 (-) | 378 (II) | 378 (-) | 378 ^np^ | 378 (III) | 378 ^np^ | 378 (I, III) |
| *Xtr100* | 459 (IV) | 460^np^ | 460 (II) | 460^np^ | 459 (-),  461 (-) | 459 (III),  462 (II) | 459 (4U),  461 (6M) | 459 (II),  462 (I) |
| *Xtr101* | 178 (IV) | 178 ^np^ | 178 (II) | 178 ^np^ | 178^np^ | 178 (II, III) | 178^np^ | 178 (I, III) |
| *Xtr102* | 318 (IV) | 318 (4U) | 305 (II) | 305^np^ | 305 ^np^,  318 (-) | 305 (-),  318 (-) | 316 (-),  318 (4U) | 305 (I),  318 (II) |
| *Xtr103* | 270 (IV) | 270 (4U) | 261 (II) | 261 (6M) | 261 (-),  270 (-) | 261 (II),  270 (III) | 261 (6M),  270 (4U) | 261 (I),  270 (II) |
| *Xtr104* | 423 (IV) | 423 (4U) | 406 (II) | 406 (6M) | 406 (-),  423 (-) | 406 (-),  423 (-) | 406 (6M),  423 (4U) | 406 (I),  423 (II) |
| *Xtr105* | 262 (IV) | 262^np^ | 279 (II) | 279 (-) | × | 262 (-),  279 (-) | 262^np^ | 262 (III),  279 (II) |
| *Xtr106* | 241 (IV) | 239 ^np^ | 239 (IV) | 239 ^np^ | 240^np^,  241 (7M) | 240 (IV) | 240^np^ | 239 (III) |
| *Xtr107* | 471 (IV) | 471 (-) | 468 (-) | 466 (-) | 471 (-) | 471 (III) | 471 (-) | 465 (III),  471 (III) |
| *Xtr110* | 362 (IV) | 362^np^ | 358 (II) | 362^np^ | 358 (-),  362^np^ | 358 (III),  362 (III) | 362^np^ | 362 (III) |
| *Xtr112* | 384 (IV) | 384 ^np^ | 390 (II) | 390 (6M) | 384 (3M),  387^np^ | 384 (III),  387 (II) | 384 (-),  387^np^ | 384 (III),  387 (I) |
| *Xtr126* | 402 (IV) | 402 ^np^ | 499 (I) | 499 (-) | 402 ^np^ | 402 (IV) | 402 ^np^ | 402 (III) |
| *Xtr128* | 214 (IV) | 214 (5U) | 197 (III) | 212 (5M) | 214 (-),  216 (-) | 212 (II),  214 (III) | 210 (5M),  214 (5U) | 214 (III) |
| *Xtr129* | 242 (IV) | 242 (4U) | 240 (I, II) | 240 (-) | 240 (2M, 3M), 242 (-) | 240 (II),  242 (III) | 242 (-),  300 (4U) | 240 (III),  300 (II) |
| *Xtr131* | 470 (IV) | 470 (5U) | 356 (I+II) | 356 (-) | 356 (2M, 3M), 464 (-) | 356 (II),  470 (III) | 396 (-),  470 (5U) | 354 (IV),  470 (III) |
| *Xtr134* | 255 (II=6U) | 255^np^ | 250 (I+II) | 250^np^ | 250 (2M, 3M), 255^np^ | 250 (II),  255 (II) | 255^np^ | 250 (IV),  255 (II) |
| *Xtr135* | 262 (I=1U) | 262 ^np^ | 260 (I) | 260 ^np^ | 260 ^np^,  262 ^np^ | 258 (I=1U),  260 (II) | 260 ^np^,  262 ^np^ | 260 (I),  262 (I) |
| *Xtr143* | 367 (IV) | 367^np^ | 367 (-) | 367^np^ | 367^np^ | 367 (III) | 367^np^ | 367 (III) |
| *Xtr146* | 303 (IV) | 303 (2U) | 381(-) | 381 (2M) | 303 (-),  381 (-) | 303 (III),  381 (-) | 303 (2U),  381 (3M) | 303 (III),  381 (IV) |
| *Xtr150* | 205 (IV) | 205^np^ | 201(III) | 201 (2M) | 201 (3U, 2M,  7M),  205^np^ | 205 (III) | 201 (7M),  205^np^ | 205 (III) |
| *Xtr154* | 229 (IV) | 229 ^np^ | 229 (IV) | 229 ^np^ | 229 ^np^ | 229 (III) | 229 (-) | 229 (III) |
| *Xtr232* | 382 (I=1U) | 382 ^np^ | 382 (II) | 382 ^np^ | 382 ^np^ | 382 (I=1U, III) | 382 ^np^ | 382 (I, II) |
| *Xtr248* | 208 (IV) | 137 (-) | 391 (II) | 391 (-) | 208 (1U) | 391(-) | 208 (5U) | 201(-) |
| *Xtr310* | 258 (IV) | 258 ^np^ | 257 (IV) | 257 (3M) | 258^np^ | 258 (III) | 258 ^np^ | 258(I, II, IV) |
| *Xtr329* | 257 (IV) | 257 (7U) | 258 (IV) | 258^np^ | 257 (-),  259^np^ | 257 (III),  258 (III) | 258^np^ | 257 (III),  258 (IV) |
| *Xtr330* | 258 (IV) | 258 ^np^ | 258 (IV) | 258 ^np^ | 258 ^np^ | 258 (II) | 258 ^np^ | 258 (IV) |
| *Xtr366* | 216 (IV),  255 (IV) | 216 ^np^,  255 ^np^ | 216 (IV),  255 (II) | 216 ^np^,  255 ^np^ | 216 ^np^,  255 ^np^ | 216 (III),  255 (III) | 216 ^np^,  255 ^np^ | 216 (IV),  255 (I) |
| *Xtr372* | 216 (II=6U) | 216 (6U) | 216 (IV) | 216 (7M) | 216 (6U, 7M) | 216 (-) | 216 (7M) | 216 (II, IV) |
| *Xtr383* | 165 (II=6U) | 165 (6U) | 183 (IV) | 183 (-) | 165 (6U, 7M), 183 (-) | 165 (-),  189 (-) | 165 (-),  186 (-) | 165 (II),  183 (IV) |
| *Xtr400* | 127 (II=6U),  147 (IV) | 147^np^ | 125 (I),  147 (IV) | 125 (-),  147^np^ | 125 (-),  127 (-),  147^np^ | 147 (IV) | 102^np^,  127 (-),  147^np^ | 147 (III, IV) |
| *Xtr413* | 277 (IV) | × | 277(IV) | × | 277 ^np^ | 277(IV) | 277 ^np^ | 277(III, IV) |
| *Xtr437* | 265 (-) | 265 (-) | 265 (-) | 265 (-) | 265 (-) | 265(III) | 265 (-) | 265 (III) |
| *Xtr451* | 262 (IV) | 192 (2U),  262 (-) | 264 (III) | 264 (2M) | 262 (-),  264 (-) | 262 (III),  264 (III) | 262 (2U),  264 (2M) | 262 (III),  264 (III) |
| *Xtr462* | 161 (IV) | 161 ^np^ | 161 (II) | 161 ^np^ | 161^np^ | 161 (III) | 161 ^np^ | 161 (III) |
| *Xtr471* | 254 (IV),  276 (IV) | 254^np^,  276^np^ | 209 (III),  270 (III, IV) | 209 (5M) | 209 (-),  254 (-),  263 (3M),  270 (-),  276^np^ | 209 (III),  254 (III),  270 (IV),  276 (III) | 170 (-),  209 (-),  254 ^np^,  276 ^np^ | 209 (III),  254 (III),  270 (IV),  276 (III) |
| *Xtr488* | 154 (IV) | 154 ^np^ | 154 (II) | 154 ^np^ | 154 ^np^ | 154 (II) | 154 ^np^ | 154 (II) |
| *Xtr537* | 254 (I=1U) | 254 (-) | 260 (I) | 260 (-) | 254 (-) | 254 (I=1U) | 254 (1U) | 254 (I) |
| *Xtr570* | 261 (IV) | 261 ^np^ | 261 (III, IV) | 261 ^np^ | 261 ^np^ | 261(III) | 261 ^np^ | 261(III) |
| *Xtr590* | 201 (I=1U)) | 201^np^ | 200 (I) | 200 (-) | 200 (-),  201^np^ | 200 (III),  201 (I=1U) | 200 (-),  201 ^np^ | 200 (II),  201 (I) |
| *Xtr615* | 204 (IV) | 205^np^ | 204 (II) | 204 (2M) | 204 (7M),  205^np^ | 204 (III, IV) | 204 (7U, 7M), 205^np^ | 204 (III),  205 (IV) |
| *Xtr641* | 248(I=1U) | 248^np^ | 247 (II) | 247(-) | 247 (2M),  248^np^ | 247(-),  248(-) | 248 ^np^ | 247(III),  248 (II) |
| *Xtr654* | 255 (IV) | 255 ^np^ | 255 (II) | 255 ^np^ | 255^np^ | 255(-) | 255^np^ | 255 (III) |
| *Xtr731* | 380 (-) | 380^np^ | 380 (-) | 380 ^np^ | 380 (-),  385 (-) | 380 (-),  385 (-) | - | 380 (-),  385 (-) |
| *Xtr757* | 187 (IV) | 187^np^, | 190 (II) | 190^np^ | 187^np^,  190^np^ | 187 (III),  190 (II) | 187^np^ | 187 (III),  190 (III) |
| *Xtr764* | 207 (IV) | 207^np^ | 214 (III) | 214 (5M) | 207^np^,  214 (-) | 207 (III),  214 (III) | 207^np^,  214 (5M) | 207 (III),  214 (III) |

n.i.: not investigated

-: amplified PCR products could not be assigned to *Aegilops* chromosomes due to the incomplete set of addition lines

np: non-polymorphic PCR product between the wheat and *Aegilops* parents of the addition lines

×: no PCR product was amplified
