# Supplementary material for: Syntenic Relationships between the U and M Genomes of Aegilops, Wheat and the Model Species Brachypodium and Rice as Revealed by COS Markers
Source: PLoS One. 2013 Aug 5;8(8):e70844. doi: 10.1371/journal.pone.0070844 (PMC3733919; doi:10.1371/journal.pone.0070844)
Supplement: Table S7 — Syntenic relationship of U and M genomes relative to wheat. (DOC) [file pone.0070844.s008.doc]

**Table S7**. Syntenic relationship of U and M genomes relative to wheat.

| *Aegilops* chromosome | Homology to D genome of wheat |
| --- | --- |
| 1U | This is mostly homologous with 1D, but two markers indicate that 1U may have segments from 2U and 4U in *Ae. umbellulata*. Similar results were found in *Ae. biuncialis* and *Ae. geniculata* (where two and one markers indicate the segments from 2U and 4U). |
| 2U | This chromosome is homologous with 2D in *Ae. umbellulata*, *Ae. biuncialis* and *Ae. geniculata*. |
| 3U | Chromosome 3U is homologous with most of 3D. One marker may indicate the presence of a segment homologous with 7D in the three U genome *Aegilops* species. Two markers may also reflect the presence of small fragments related to 6D and 5D in *Ae. umbellulata* and *Ae. biuncialis*, respectively. |
| 4U | A very small number of markers (2, 1 and 2 in *Ae. umbellulata*, *Ae. biuncialis* and *Ae. geniculata*, respectively) may indicate homology with 6D. |
| 5U | Chromosome 5U is homologous with 5D, but a small segment from 2U might be present. |
| 6U | This study indicated the highly rearranged nature of chromosome 6U, as it contains segments homologous with 2D, 4D and 7D in the three U-genome *Aegilops* species. Two markers may also reflect the presence of a small segment related to 5D in the diploid *Ae. umbellulata*. |
| 7U | The involvement of this chromosome in a 7U-3U rearrangement is indicated by markers specific for 7D and 3D in the three *Aegilops* species. A small segment from 6U might be indicated by one marker in *Ae. geniculata*. |
| 1M | A very small number of markers indicated that chromosome 1M is homologous with 1D and 2D. |
| 2M | The homology of this chromosome with 2D was indicated in *Ae. comosa, Ae. biuncialis* and *Ae. geniculata*. |
| 3M | Similarly to 3U, this chromosome is homologous with 3D. Two markers may indicate the presence of small segments from 5M and 1M in *Ae. comosa* and *Ae. biuncialis*, respectively. |
| 5M: | This chromosome is homoeologous with 5D in the three M-genome *Aegilops* species. |
| 6M | Similarly to 6U, the chromosome 6M might also be rearranged. The involvement of 6M in a 6M-7M rearrangement is indicated by markers specific for 6D and 7D in the three *Aegilops* species. The presence of small segments homologous with 1D and 4D was indicated by one and two markers, respectively, in *Ae. comosa*. |
| 7M | The involvement of this chromosome in a putative 6M-7M rearrangement is also indicated in the three M-genome species. Small segments from 2M and 5M might be present in *Ae. geniculata*. |
